# Minimum tillage as climate-smart agriculture practice and its impact on food and nutrition security

**DOI:** 10.1371/journal.pone.0287441

**Published:** 2023-12-22

**Authors:** Baba Adam, Awudu Abdulai

**Affiliations:** Department of Food Economics and Food Policy, Institute of Food Economics and Consumption Studies, Kiel University, Kiel, Germany; Hunan Agricultural University, CHINA

## Abstract

Minimum tillage (MT) is a sustainable farming practice that limit soil disturbance only to planting stations while leaving the rest of the soil undisturbed. It is an important component of conservation agriculture, which aims to raise agricultural productivity, improve the livelihoods of farmers and build resilient farming systems. Despite the growing empirical literature on its adoption and benefits, there is a paucity of empirical evidence on the heterogeneous effect of length of MT adoption on household welfare. This study uses plot-level and household data combined with geo-referenced historical weather data to provide microeconomic evidence of the impact of MT on maize yields, food and nutrition security, and farm labor demand in Ghana. We account for potential selection bias and omitted variable problems by using an ordered probit selection model to estimate two transition-specific treatment effects: from conventional tillage systems to short-term MT adoption and from short-term to long-term MT adoption. The empirical results show that longer cropping seasons of MT adoption significantly increases maize yields and dietary diversity by about 4.33% and 14.22%, respectively, and decreases household food insecurity and labor demand by 42.31% and 11.09%, respectively. These findings highlight the necessity of developing and implementing programs that promote and help smallholder farmers to sustain its adoption for longer cropping season.

## Introduction

Soil degradation as one of the global environmental challenges is happening at an alarming rate and poses a major threat to crop productivity worldwide. Intensive conventional tillage systems such as ploughing, ridging, and harrowing, leave the soil surface bare and loosen soil particles, causing high levels of soil surface runoff and erosion as well as inhibit the buildup of soil organic matter [1, 2]. Available evidence indicates that an estimated 25 percent of the world’s total land area have been degraded while 24 billion tons of fertile soils are lost every year, partly due to excessive soil tillage and other unsustainable land-use practices [3].

According to the United Nations Convention to Combat Desertification (UNCCD), approximately two-thirds of arable lands in sub-Saharan Africa are subjected to soil degradation due to unsustainable conventional farming practices, which contribute to soil compaction, soil erosion, and depletion of soil organic matter, affecting at least 485 million smallholder farmers. [4]. At the same time, food production systems need to sustainably raise crop productivity to meet the growing food demand [5]. Sustainable farming practices that contribute to stabilizing food production while improving soil organic matter content and increasing carbon sequestration in biomass and soils have to be promoted [6, 7].

There is widespread recognition among policy-makers that achieving the SDG on Zero Hunger by 2030 may be in jeopardy unless concerted efforts are made to stabilize food production through a shift to sustainable farming practices [5, 8]. Agricultural policies and programs have over the past decades focused on promoting conservation agriculture (CA) as an option to improve soil fertility and increase crop productivity. Conservation agriculture comprises a set of crop management principles, namely, minimum soil disturbance (e.g., minimum tillage), maintenance of permanent soil cover, and rotation or association with diverse crops [9]. Minimum tillage (MT) causes minimum soil disturbance save the planting area. The technology offers a clear pathway to increase crop productivity by optimizing input use, reducing labor demands during peak season, increasing soil organic matter content, preventing soil erosion, and improving infiltration of rainfall water [10, 11].

Notwithstanding the aforementioned benefits, minimum tillage adoption remains low, coupled with increased dis-adoption among smallholder farmers [8, 12, 13]. This calls into question its effectiveness in sub-Saharan Africa [14]. One of the major trade-offs that potentially accounts for this low adoption is that productivity gains remain uncertain and controversial [14–16]. While some studies have observed immediate yield gains from the adoption of minimum tillage and other CA practices [12, 17], others observed a lag period of 2–5 cropping seasons or even longer periods before any significant yield gains [14, 18]. For instance, in Zimbabwe, Michler et al. [19] found that in the short term, that is, within four years, the adoption of minimum tillage had either a negative impact or no yield benefits on cereal production. Similarly, Thierfelder and Wall [20] observed that the adoption of minimum tillage and crop residue retention had no impact on maize yields in the first three years of adoption in Zambia.

Transitioning from conventional tillage into minimum tillage systems could have short-term benefits, with benefits accruing to adopters within the first few cropping seasons or long-term benefits, with benefits becoming visible only after several cropping seasons [14, 21, 22]. Furthermore, medium to long-term adoption of minimum tillage is expected to improve soil fertility and productivity over time, due to the gradual improvements in the biological, physical, and chemical properties of the soil [23]. Thus, understanding the inter-temporal adoption decisions of smallholder farmers and the impacts of adoption are crucial in formulating targeted policies aimed at addressing their constraints to resource use [24].

In the context of increased minimum tillage promotion and continued uncertainty over its effectiveness in smallholder farming systems [15, 24], the paucity of empirical evidence on the heterogeneous effect of length of its adoption on smallholder production systems is not trivial. The bulk of the evidence is mainly based on field experiments (either on-farm or on-station demonstration fields) [e.g. 17, 20]. However, these studies may not capture the performance of the technology under heterogeneous smallholder farmers’ socio‐economic characteristics and site-specific conditions (such as soil type, climate, management practices, and topography).

This study attempts to address some of the empirical gaps and contributes to the literature on minimum tillage adoption as follows: First, we determine the impact of minimum tillage adoption transitions on maize yields, household dietary diversity, and household food insecurity access scores (HFIAS). We explore the temporal dimension of adopting minimum tillage by estimating two transition-specific treatment effects: one from conventional tillage to short-term minimum tillage adoption and from short-term to long-term minimum tillage adoption. Previous studies have analyzed the productivity and food security benefits of minimum tillage adoption [e.g. 13, 25]. However, they tend to typically estimate homogenous effects and fail to account for important treatment effect heterogeneity regarding farmers’ adoption transitions. Thus, the inability to capture the trade-off between short-term and long-term welfare benefits may lead to misleading conclusions and tend to affect its widespread adoption [26].

Second, the issue of increased labor demand under minimum tillage systems also emerges as an important point of concern. Transitioning from conventional tillage may lead to an increase in labor demand for weeding in the short-term. Thus, this increase in labor demand offsets the labor-saving gained from minimum tillage, especially in cases where herbicides are not applied [12]. However, maintaining a protective layer of crop residues on the soil surface may lead to economic benefits such as reduced total labor demand in the long-term [14]. Thus, understanding the potential magnitude of the impact of labor requirements, and taking into account the heterogeneity in the length of adoption is essential to properly evaluate the range of benefits and potential trade-offs of minimum tillage adoption.

To the best of our knowledge, this paper offers an empirically sound analysis on the impact of minimum tillage adoption under typical smallholder conditions in sub-Saharan Africa. We accounts for potential threats of selection bias and omitted variable problems by employing the ordered-probit selection model to distinguish between conventional tillage system, short-term, and long-term adoption of minimum tillage. We use recent survey data of maize farm households from the northern savanna zone of Ghana and geo-referenced historical weather data (rainfall and temperature) (1981–2018) to control for the effect of climatic shocks.

## Materials and methods

### Ethics statement

This study was approved by the Chair of Food Economics and Food Policy, Department of Food Economics and Consumption Studies, University of Kiel. Verbal consent was obtained from each study participant.

### Farm household survey

The data come from 489 maize farm households in the northern Savanna zone of Ghana. A survey was conducted for the 2018/2019 cropping season in 15 communities across six districts and three regions under the Sustainable Land and Water Management Project (Funded by World Bank through the Global Environment Facility) intervention areas. We purposively selected six districts from the three regions based on the operational areas of the project and the prevalence of minimum tillage technologies. Further, we randomly selected three to six communities from each district. Finally, 489 maize farm households operating approximately 886 maize plots were randomly sampled in proportion to the farmer population in the community. The survey data were collected using a structured questionnaire by trained and qualified researchers and enumerators who have good working knowledge of the farming systems in the study areas. The survey also collected household and plot-level data on production costs, prices, household demographic characteristics, access to market information, extension contacts, and minimum tillage practices being implemented, as well as the food security situation of farm households.

Following previous literature on conservation agriculture [e.g., 15, 19, 25, 27], we present in Table 1 a detailed description of the control variables used in our empirical analysis. Concerning household-level information and demographic characteristics, we control for age, gender, education of household head, household size, and asset ownership (e.g., farm size, livestock ownership). We also control for information access (extension service), membership of Farmer Based Organizations (FBO), and resource constraints (credit access). Based on the approach of Abdulai and Huffman (2014), we define credit-constrained farmers as those who received credit but expressed a desire to borrow more at the existing interest rate, those whose credit request was denied, or those who had no access to any form of credit. Our analysis also accounts for plot-level characteristics such as soil fertility (fertile and moderately fertile), slope, and control for location, differentiating between the regions used for the study to account for location fixed effects.

**Table 1:** Variable descriptions and descriptive statistics.

Variable	Description	Mean	Std. Dev
**Outcome variables**			
Maize yields	The gross value of production metric ton (hectares)	1.961	1.322
HFIAS	Household food insecurity assess scores, where 0 represents a food secure HH, and 27 a food-insecure HH.	4.37	2.67
Dietary diversity	Simpson index (based on calorie share).	0.72	0.14
Labor demand	Total person-days (hectares)	58.54	57.47
**Treatment variables**			
Long-term MT adoption	1 indicates a continuous MT adoption for more than 4 cropping seasons, 0 otherwise	0.315	0.46
Short-term MT adoption	1 indicate a continuous MT adoption for less than 4 cropping seasons, 0 otherwise	0.262	0.44
Conventional Tillage (CT)	1 if HH practice CT on plot, 0 otherwise	0.423	0.49
**Explanatory variables**			
Age	Age of household head in years	42.35	12.89
Male headed	Male = 1, Female = 0	0.63	0.48
HH size	Size of Household	5.97	2.97
Education	Years of formal education	3.72	4.77
FBO	1 if farmer belongs to farmer-based organization, 0 otherwise	0.31	0.46
Farm size	Total farm size of plot (hectares)	1.19	1.08
Extension	Number of extension contacts per annum	10.35	6.91
Credit access	1 if farmer credit constrained, 0 otherwise	0.36	0.48
Livestock	Number of Livestock in Tropical Livestock Units	1.82	4.12
Mean_slope	Perception that plot has is moderately sloped (1 = yes; 0 = no)	0.66	0.47
Mean_fertile	Perception that plot has is fertile (1 = yes; 0 = no)	0.37	0.48
Mean_Mod. fertile	Perception that plot is moderately fertile (1 = yes; 0 = no)	0.51	0.50
Asset	Value of HH durable assets (`000 GHS)	2.53	2.46
Negative rainfall shock	1 if the metrics indicate a level of rainfall with a standard deviation below the long-term mean, 0 otherwise	0.47	0.50
Mean temperature	Mean temperature in ⁰C (1981 to 2018)	27.33	0.47
Fertilizer	Total monetary value of fertilizer used (GHS) (hectares)	311.27	284.36
Climate information	1 if HH received climate information prior to adoption; 0 otherwise	0.60	0.49
CA Training	Number of training received on conservation agriculture (CA)	3.55	3.33
Northern region	Northern region = 1, 0 otherwise	0.37	0.48
Upper East	Upper East region = 1, 0 otherwise	0.32	0.47
Upper West	Upper West region = 1, 0 otherwise	0.31	0.46

MT: Minimum tillage

In addition, we include climatic variables to show differences in seasonal mean temperature and rainfall and their influence on farmers`choices concerning the length of minimum tillage adoption. Variability in the frequency and intensity of rainfall, rising mean temperatures, and seasonal dry spells have adverse effects on food production and threaten the livelihoods of resource-poor farmers [19, 28, 29]. To control for the impact of rainfall shocks and increased temperatures on yields and food and nutrition security, we use rainfall and temperature data (from the Prediction of Worldwide Energy Resources data archive) (https://power.larc.nasa.gov/data-access-viewer) that provides global data at high-resolution (0.5x0.5 degree) to generate a gridded rainfall and temperature time series. We use household geographical coordinates to derive long-term mean rainfall and temperature data (from 1981–2018) for the months within the cropping season (i.e. April to October). Thus, following previous literature [19, 30], we compute rainfall shocks using a dummy variable to capture the differential exposure to rainfall (negative rainfall shock) as follows:

${\underline{RS}}_{kt}=|\frac{R_{kt}-{\bar{R}}_{k}}{\sigma_{R_{k}}}|$ if $R_{kt}<{\bar{R}}_{k}$, 0 otherwise, where *R*_*kt*_ is the

Based on the literature and our study context, this paper defines minimum tillage as a technique with low soil disturbance, where tillage is restricted to planting stations and the remaining soil is left undisturbed. These practices include Zai technique, ripping, and zero tillage. The Zai technique is a traditional name for planting basins made with hand-hoes, where seeds are sown in small planting pits and filled with organic material such as crop residues [31]. This practice reduces soil erosion, improves microbial activities, and concentrates soil water around the base of the plant, thereby supporting plant growth during seasonal dry spells. Ripping is a minimumtillage technique where rip lines are constructed with ox- or tractor-drawn rippers, and zero tillage with jab planters or dibble sticks [15]. Due to the minimum soil disturbance, crop residues from previous seasons harvests are usually left on the surface of the soil. The non-adopters comprise all other farmers who used conventional tillage practices including ploughing, ridging, harrowing, and hand hoeing. To measure the time length of adoption, we construct our treatment variable into three different adoption states: conventional tillage, short-term minimum tillage adoption, and long-term minimum tillage adoption to provide better insights into its potential economic benefits.

**Fig 1:**
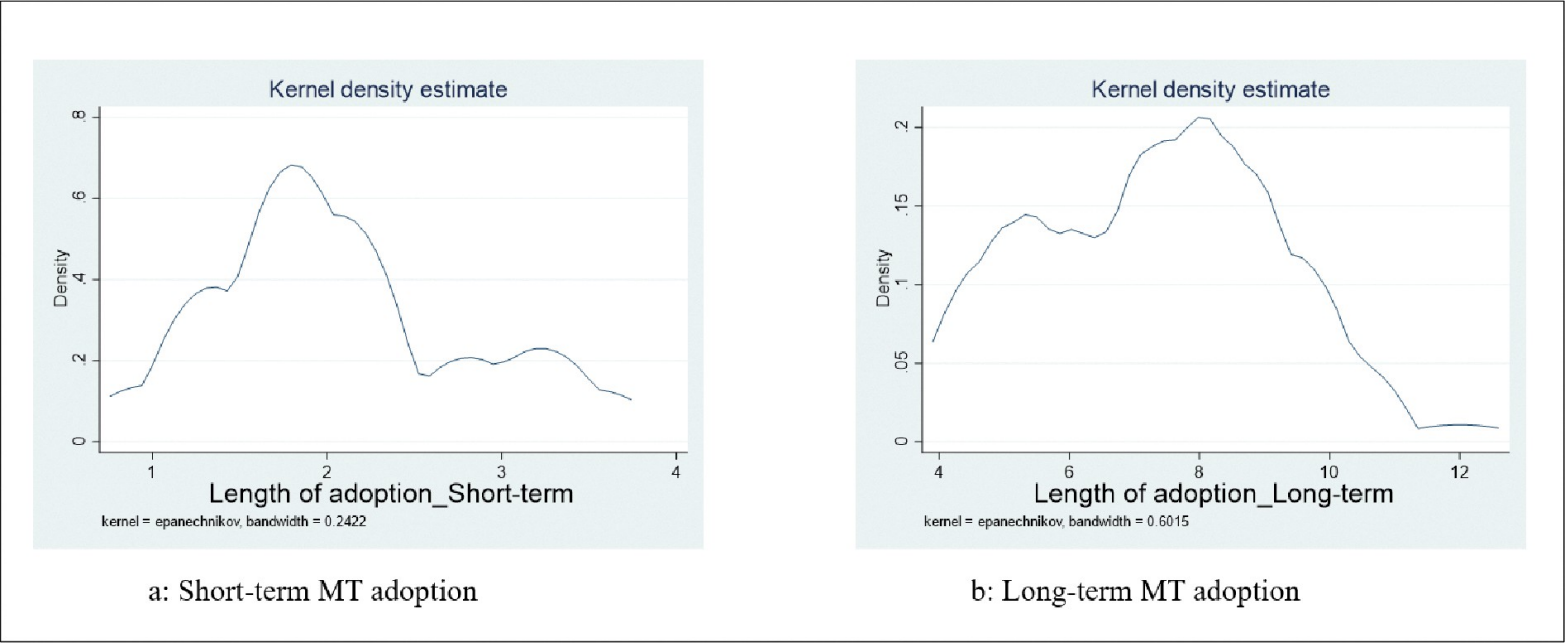
Distribution of the length of minimum tillage adoption. 1a: Short-term MT adoption, lb: Long-term MT adoption.

We constructed a dynamic adoption data set based on recall data and information from our cross-sectional survey. Smallholder farmers’ recall information can be used to approximate the dynamic pattern of adoption in the absence of longitudinal data [32]. Farmers were asked when they first heard about the technology and when they first used it on their farms. Accordingly, we define short-term adoption to include plots where minimum tillage has been practiced for less than 4 cropping seasons, and medium- to long-term (henceforth referred to as “long-term”) for those where minimum tillage has been adopted for more than 4 cropping seasons. Fig 1 presents the kernel density estimates of the length of adoption. It is clear from the figure that the majority of short-term adopters are within 1–2 years of adoption, while the majority of long-term adopters are within 7–10 years of adoption. This distribution further indicates the importance of analyzing the heterogeneity in the length of adoption with respect to the expected welfare outcomes.

*Measurement of food and nutrition security*. Two important indicators of food and nutrition security are employed in the study: household dietary diversity and household food insecurity access scores (HFIAS). We compute the dietary diversity of households using the Simpson’s index (SI) of dietary diversity. This measure is a good proxy for a household’s socio-economic capacity to access a variety of foods. Understanding dietary diversity is vital because nutritional levels differ between food items and food groups. A number of empirical studies [33, 34] have measured dietary diversity using a count of all food groups consumed by the household. However, this approach assigns equal weights to the food groups or items regardless of the calorie and nutrition content and hence is unable to provide sufficient information on household dietary diversity. Therefore, to overcome this problem, we convert the quantity of food groups consumed into calories using food composition tables for Western Africa developed by FAO [35] to account for the number of food items consumed and the respective magnitude (calories) of each food group [36, 37].

We calculate the calorie consumption using the following food items: (i) Cereals, (ii) roots and tubers, (iii) pulses (iv) oils and fats, (v) vegetables, (vi) fruits, (vii) meat (viii) egg, (ix) fish, (x) dairy products, (xi) legumes, nuts and seeds, (xii) sweets and (xiii) beverages [38, 39]. Mathematically, Simpson index can be computed as follows:

$$SI=1-\sum_{i-1}^{n}{w_{i}}^{2}$$

where *w*_*i*_ is the calorie share of food item in the total amount of calories consumed. The Simpson index is in the range of zero to one. Thus, a higher index signifies better dietary diversity.

In addition, we measure household food insecurity access scores to capture different behavioral and psychological dimensions of food security [40]. HFIAS comprises nine occurrence questions on the prevalence of food insecurity (access) with three levels of severity based on a recall period of the past four weeks (30 days). The maximum score for a household is 27, representing poorer access to food and greater household food insecurity; the minimum score is zero (0), which signifies that the household is food secure [39, 41].

Table 1 presents the definitions and the descriptive statistics of the selected variables used in the analysis. The sample average household size is six persons with a mean farm size of approximately two hectares. The average level of education is approximately 3.72 years of schooling, indicating lower years of education across the study areas, compared to the national average of 7.3 years [29, 42]. Table 2 also presents the mean differences in the outcome variables and household characteristics across minimum tillage adoption status.

**Table 2:** Mean differences in characteristics.

Variable	Conventional tillage			Short-term MT adoption			Long-term MT adoption	
	1		Diff. (t-stat.)	2		Diff. (t-stat.)	3	
	Mean	Std. Dev.	(2–1)	Mean	Std. Dev.	(3–2)	Mean	Std. Dev.
**Outcome variables**								
Maize yields	1.43	2.20	0.71***	2.14	1.78	0.31**	2.45	1.71
HFIAS	5.54	2.44	-0.72***	4.42	2.45	-2.42***	2.40	1.99
Dietary diversity	0.61	0.004	0.09***	0.70	0.01	0.18***	0.89	0.003
Labor demand	62.37	47.56	5.32***	67.69	94.94	-22.42***	45.27	48.22
Hired labor	21.50	17.01	20.69***	42.19	29.32	-19.08***	23.11	12.22
Family labor	40.87	32.54	-15.37**	25.50	17.14	-3.34**	22.16	13.15
**Explanatory variables**								
Age	41.09	13.22	1.52	42.61	12.15	0.81	43.42	12.46
Male headed	0.44	0.50	0.29***	0.73	0.44	0.06	0.80	0.40
HH size	5.95	3.25	-0.07	5.88	2.79	0.19	6.08	2.73
Education	2.65	3.87	1.80***	4.45	5.12	0.10	4.56	5.29
FBO	0.23	0.42	0.07	0.30	0.46	0.11**	0.41	0.49
Farm size	1.32	1.25	-0.35***	0.97	0.74	0.21**	1.18	1.02
Extension	8.40	6.36	1.57**	9.98	5.59	3.30***	13.28	7.62
Credit access	0.46	0.50	-0.09*	0.37	0.48	-0.13**	0.23	0.42
Livestock	1.22	3.32	0.95**	2.17	4.21	0.14	2.31	4.87
Slope	0.68	0.47	-0.001	0.68	0.47	-0.07	0.61	0.49
Fertile soil	0.38	0.48	0.03	0.40	0.49	-0.05	0.36	0.48
Moderately fertile soil	0.45	0.50	0.08	0.53	0.50	0.02	0.55	0.50
Asset (log)	6.74	1.14	0.61***	7.36	0.07	0.58***	7.94	0.06
Negative rainfall shock	0.34	0.47	0.18***	0.52	0.50	0.07	0.59	0.49
Mean temperature	27.29	0.46	0.07	27.37	0.48	-0.02	27.34	0.48
Fertilizer	208.13	123.67	79.73***	128.39	91.50	2.47	130.87	95.73
Climate information	0.36	0.48	0.46***	0.83	0.38	-0.09*	0.73	0.44
CA Training	0.71	1.14	4.17***	4.88	3.09	0.13	5.01	2.82
No. of obs. (plots)	391			226			269	

Note: Significance level at

*p < 0.1

**p < 0.05

***p < 0.01

Exchange rate at the time of survey: 1 USD = 5.14 Ghana Cedis (GHS)

In particular, we find that long-term adopters have significantly higher maize yields (2.45 Mt/ha) compared to short-term adopters (2.14 Mt/ha) and conventional tillage farmers (1.43 Mt/ha). Also, the average HFIAS is lower for long-term adopters (2.40) compared to short-term adopters (4.42) and conventional tillage farmers (5.54), signifying that long-term minimum tillage adoption plays a significant role in improving the food security situation of households. Similarly, the average Simpson index value is higher for long-term adopters (0.89), compared to short-term adopters (0.70) and conventional tillage farmers (0.61), indicating a high level of dietary diversity for long-term adopters.

Concerning the total labor demand, short-term adopters use more labor (67.69 person-days/ha) compared to long-term adopters (45.27person-days/ha) and conventional tillage farmers (62.37 person-days/ha). This result could be explained by the relatively large amount of labor required for manual weed control during this stage of adoption (see Table A2 in S1 Appendix). Generally, the estimates vary significantly between conventional tillage farmers, short-term adopters, and long-term adopters for some household and farm-level characteristics and outcome variables. However, these effects may not signify the impacts of adoption, as these comparisons do not take into account confounding factors that may affect the adoption of minimum tillage.

### Conceptual framework

We consider the minimum tillage adoption decisions as a constrained optimization problem where farm households’ transition into different minimum tillage adoption states depends on several factors including available information, relative costs and benefits of minimum tillage, and other socio-economic conditions. Given these constraints smallholder farm households may decide to transition into minimum tillage adoption (i.e., short-term or long-term), if the expected net benefits from transitioning are higher than the expected net benefits of conventional tillage (CT) as follows, i.e. when $\pi_{i}^{*MT}>\pi_{i}^{*CT}$ [49]:

$$\pi_{i}^{*MT}=p_{i}Q_{i}^{*MT}-\sum_{j=1}^{J}{\omega_{ji}X}_{ji}^{*MT}$$

$$\pi_{i}^{*CT}=p_{i}Q_{i}^{*CT}-\sum_{j=1}^{J}{\omega_{ji}X}_{ji}^{*CT}$$ (1)

Where $Q_{i}^{*MT}$ and $Q_{i}^{*CT}$ are vectors of maize yields for minimum tillage and conventional tillage farmers; *p*_*it*_ is a vector of maize prices which is assumed to be the same for both categories of farmers; *X*_*ji*_ and *ω*_*ji*_ are vectors of input quantity and input prices; respectively.

In order to comprehend how minimum tillage affects household welfare, we consider the various pathways through which transitioning into minimum tillage can potentially influence food and nutrition security, as well as labor demand. Transitioning into minimum tillage adoption would affect food and nutrition security primarily through an increase in crop productivity (maize yields) and income. Higher crop productivity can be realized from the agronomic benefits of minimum tillage such as increased soil moisture, enhanced input use efficiency, and increased soil organic matter [10, 12, 43]. The resulting improvement in yields may increase household food availability from own production and contribute to relaxing household consumption constraints. However, some authors [44] have argued that in some drier regions, conservation tillage is mainly employed to reduce soil degradation that is usually associated with traditional tillage systems as well as conserve soil moisture and reduce production costs rather than aiming at short-term productivity gains. Thus, whether minimum tillage contributes to higher yields is an empirical issue.

Increased household income also enhances the purchasing power of households to access diverse and micronutrient-rich foods, and thus plays an important role in enhancing their dietary diversity [45]. Furthermore, switching into long-term minimum tillage adoption is expected to save time and labor (especially critical planting time) [46] primarily due to reduced ploughing and suppression of weeds through crop residue retention. Thus, the labor saved could be channeled into a variety of income-generating activities that could potentially increase income and purchasing power of households [47]. However, it is important to note that, switching into short-term minimum tillage adoption may lead to increased labor demand for weeding if herbicides are not applied, and may reduce household income and affect consumption patterns [10, 14].

### Empirical strategy

As stated earlier, we categorize minimum tillage adoption into multiple adoption states: conventional tillage (*j* = 1), short-term adoption (*j* = 2), and long-term adoption (*j* = 3) based on the length or duration of adoption. Thus, farm households could transition from conventional tillage into short-term minimum tillage adoption or from short-term into long-term minimum tillage adoption. Given the multivalued nature of the treatment, we model minimum tillage technology adoption as an ordered choice [48–50] under the assumption that farmers are risk-neutral and take into consideration the expected benefits to be derived from adoption. However, we only observe the decision to use minimum tillage technology either in the short-term or long-term or to practice conventional tillage. We define the expected benefits which cannot be observed as a latent variable $D_{ij}^{*}$ denoting sorting of farmer *i* into the three adoption states (*j* = 1,2,3) of minimum tillage adoption, based on an ordered-probit selection rule as:

$$D_{ij}^{*}=\alpha_{j}^{\prime }Z_{ij}+\theta {\bar{Z}}_{ij}+\mu_{ij},\;\mathrm{where}$$

$$D_{ij}=1[C_{j}(w_{j})<\alpha^{\prime }Z_{i}+\theta {\bar{Z}}_{i}+\mu_{i}\leq C_{j+1}(w_{j+1})],j=1,2\ldots \bar{J}$$ (2)

and the cutoff values satisfy

$$C_{j}(w_{j})\leq C_{j+1}(w_{j+1}),\;C_{0}(w_{0})=-\infty ,\;and\;C_{\bar{J}}(w_{\bar{J}})=\infty$$

in which *D*_*ij*_ is a multivalued treatment variable, ***Z***_*i*_ is a vector of observed variables, ${\bar{Z}}_{i}$ is mean plot-level characteristics, $\alpha^{\prime }Z_{i}+{\bar{Z}}_{i}+\mu_{i}$ is a latent linear index, *α* is a vector of estimated parameters, *w*_*j*_ is a vector of observed regressors, *C*_*j*_(*w*_*j*_) are threshold parameters, which are allowed to depend on the regressors, and *μ*_*ij*_ are error terms.

We express our observed outcomes *Q*_*ij*_, as a linear function of a vector of *X*_*i*_ for each of the adoption state *j* = (1,2,3) as follows:

$$Q_{ij}=\left\{ \begin{array}{c} \beta_{1}X_{i}+\theta_{1}{\bar{X}}_{i}+\varepsilon_{i1}\;if\;D_{i}=1\\ \beta_{2}X_{i}+\theta_{2}{\bar{X}}_{i}+\varepsilon_{i2}\;if\;D_{i}=2\\ \beta_{3}X_{i}+\theta_{3}{\bar{X}}_{i}+\varepsilon_{i3}\;if\;D_{i}=3 \end{array}\right.$$ (3)

where the parameter vector, *β*_*j*_, of *X*_*i*_ depend on the treatment state *j*, *D*_*i*_ is the choice of the length of adoption, and *ε*_*i*_ has a zero mean and has a variance of $\sigma_{j}^{2}$, for each *j* = 1,2,3.

Given that the adoption of minimum tillage is not random, unobserved traits (such as skills, ability, and motivation) that would likewise correlate with the outcome variables of interest might have an impact on smallholder farmers’ decisions, leading to issues with self-selection and omitted variables.

Furthermore, there may be heterogeneity in returns to minimum tillage adoption such that smallholder farmers who anticipate higher returns to minimum tillage are most likely to adopt [27, 51]. Thus, the estimation of specification (3) with the OLS approach could lead to biased and inconsistent estimates. We address this potential bias employing the ordered-probit selection model [33, 34]. This model uses full information maximum likelihood (FIML) to simultaneously estimate selection Eq (2) and the outcome specification (3) for the three adoption states. We further correct the selection bias and omitted variable problem by including the inverse mills ratios (IMR) from a first-stage ordered choice model (Eq 1) in the second-stage outcome models (Eq 3). Further, we define the correlations of these errors as *ρ*_*j*_ = Corr (*ε*_*ij*_, *μ*_*ij*_), assuming joint normality of the errors in Eqs 2 and 3. The substantive outcome equations can be rewritten in terms of expectations as;

$$E[\varepsilon_{i1}|D_{i}=1]=E[\varepsilon_{i1}|{(C_{1}(w_{1})-\alpha^{\prime }Z_{i}-\theta {\bar{Z}}_{i})<\mu }_{i}]=\rho_{1}\frac{\phi (C_{1}(w_{1})-\alpha^{\prime }Z_{i}-\theta {\bar{Z}}_{i})}{1-\Phi (C_{1}(w_{1})-\alpha^{\prime }Z_{i}-\theta {\bar{Z}}_{i})}=\rho_{1}\lambda_{1},$$

$$E[\varepsilon_{i2}|D_{i}=2]=E[\varepsilon_{i2}|(C_{2}(w_{2})-\alpha^{\prime }Z_{i}-\theta {\bar{Z}}_{i})<\mu_{i}\leq (C_{1}(w_{1})-\alpha^{\prime }Z_{i}-\theta {\bar{Z}}_{i})]$$

$$=\rho_{2}\frac{\phi (C_{2}(w_{2})-\alpha^{\prime }Z_{i}-\theta {\bar{Z}}_{i})-\phi (C_{1}(w_{1})-\alpha^{\prime }Z_{i}-\theta {\bar{Z}}_{i})}{\Phi (C_{1}(w_{1})-\alpha^{\prime }Z_{i}-\theta {\bar{Z}}_{i})-\Phi (C_{2}(w_{2})-\alpha^{\prime }Z_{i}-\theta {\bar{Z}}_{i})}=\rho_{2}\lambda_{2},$$ (4)

$$E[\varepsilon_{i3}|D_{i}=3]=E[\varepsilon_{i3}|\mu_{i}\leq (C_{2}(w_{2})-\alpha^{\prime }Z_{i}-\theta {\bar{Z}}_{i})]=\rho_{3}\frac{-\phi (C_{2}(w_{2})-\alpha^{\prime }Z_{i}-\theta {\bar{Z}}_{i})}{\Phi (C_{2}(w_{2})-\alpha^{\prime }Z_{i}-\theta {\bar{Z}}_{i})}=\rho_{3}\lambda_{3},$$

The ratios on the right-hand side of Eq (4) are the Heckman-type selection correction terms, which are constructed based on predicted probabilities from the first-stage selection Eq (2) and included as correction terms in the outcome equations with *ρ*_1_, *ρ*_2_ and *ρ*_3_ as the associated coefficients [33, 56];

$$Q_{ij}=\left\{ \begin{array}{c} \beta_{1}X_{i}+\theta_{1}{\bar{X}}_{i}+\rho_{1}\lambda_{1}+\eta_{i1}\;if\;D_{i}=1\\ \beta_{2}X_{i}+\theta_{2}{\bar{X}}_{i}+\rho_{2}\lambda_{2}+\eta_{i2}\;if\;D_{i}=2\\ \beta_{3}X_{i}+\theta_{3}{\bar{X}}_{i}+\rho_{3}\lambda_{3}+\eta_{i3}\;if\;D_{i}=3 \end{array}\right.$$ (5)

Note that the statistical significance of the correlation coefficients (*ρ*_1_, *ρ*_2_ and *ρ*_3_) indicates the presence of selection bias. A positive sign of the *ρ*_*j*_s indicates reverse selection on unobserved gains, while a negative sign indicates positive selection on unobserved gains.

*Estimating treatment effects*. Using Eq 5, we express the treatment effects, that is, ATE^†^ (average treatment effect for the entire population), ATE (average treatment effect for those at one of the transition stages), TT (average treatment on the treated, or the gains for those who choose to transition), and TUT (average effect of treatment on the untreated, or the gains for those who chose not to transition) for farmer’s transition between *j* and *j* + 1 as follows:

$${\mathrm{ATE}}^{†}{}_{j+1,j}=E[Q_{ij+1}-Q_{ij}]$$ (6A)

$$ATE_{j+1,j}=E[Q_{ij+1}-Q_{ij}|D_{i}\in \{j,j+1\}]$$ (6B)

$$TT_{j+1,j}=E[Q_{ij+1}-Q_{ij}|D_{i}=j+1]$$ (6C)

$$TUT_{j+1,j}=E[Q_{ij+1}-Q_{ij}|D_{i}=j]$$ (6D)

The difference between ATE^†^ and ATE shows the difference in the characteristics of those in the entire population and that of those at the transitions between two adoption states. The difference between the TT and ATE measures sorting on gains, while the difference between TUT and ATE measures sorting losses. Further, the relationship between Eqs (6B) to (6D) illustrates whether patterns of heterogeneity reflect positive (TT >ATE >TUT) or reverse selection (TUT >ATE>TT) on gains. In other words, these patterns show whether economic gains are greater among farm households most or least likely to transition into short-term or long-term adoption [50, 52].

We use the Mundlak’s approach to address a potential plot-level unobserved heterogeneity problem that could arise due to farm households cultivating multiple plots [53]. Furthermore, to ensure identification and exclusion restriction, we need to have instruments that directly affect the selection equation, but not the outcome variables of interest [49]. Intuitively, one requires an instrument (as a source of variability) for each transition [48]. In our analysis, the three ordered choices imply two transitions (i.e., conventional tillage to short-term minimum tillage adoption, and short-term to long-term minimum tillage adoption) and thus requires at least two instruments.

Following previous studies [e.g., 34, 54], we use past climate information and training (i.e. average village-level training days) on conservation agriculture received by farmers as the excluded instruments. Access to reliable and timely climate information (through print and broadcast media etc.) on rainfall and temperature forecasts tend to influence behavioral changes towards the adoption of minimum tillage [55]. Similarly, training is effective in driving the adoption of conservation agriculture technologies. Increased training assists farmers in their day-to-day operations and is essential for accelerating and sustaining the adoption of innovations.

Following Di Falco et al., [54], we establish the admissibility of the instruments by carrying out a falsification test. A variable may be a valid instrument if it affects minimum tillage adoption decisions, but not the outcome variable of interest among farmers who did not adopt minimum tillage. Our results indicate that the variables are jointly significant in affecting minimum tillage adoptions (see Table 3), but not the outcome variables (see Table A1 in S1 Appendix). A further test of correlation in Table A3 in S1 Appendix shows that the instrumental variables are not correlated with the outcome variables of interest (i.e., the exclusion restriction assumption).

**Table 3:** First stage ordered selection model.

Variables	Conventionaltillage		Short-term MT adoption		Long-term MT adoption	
	1		2		3	
	Marginal effects	S.E.	Marginal effects	S.E.	Marginal effects	S.E.
Age	-0.001	0.002	-0.002	0.002	0.004*	0.002
Male headed	-0.144***	0.054	0.128*	0.067	0.016	0.051
HH size	-0.001	0.009	0.008	0.012	-0.007	0.009
Education	0.008	0.005	-0.012**	0.006	0.042	0.004
FBO	-0.211***	0.055	0.170***	0.061	0.058	0.046
Farm size	0.029**	0.013	-0.030**	0.014	0.001	0.010
Extension	-0.008*	0.004	-0.001	0.005	0.007*	0.004
Credit access	-0.038	0.050	0.151**	0.060	-0.112**	0.048
Livestock	-0.006	0.005	0.007	0.007	-0.001	0.005
Mean_slope	0.148***	0.054	0.001	0.063	-0.150***	0.045
Mean_fertile	-0.179**	0.083	0.192**	0.096	-0.013	0.079
Mean_Mod. fertile	-0.188**	0.082	0.152	0.096	0.036	0.078
Asset (log)	-0.109***	0.023	-0.045*	0.027	0.154***	0.022
Negative rainfall shock	-0.190***	0.069	0.275***	0.088	-0.085	0.079
Mean temperature	0.205***	0.067	-0.027	0.081	-0.178***	0.056
Fertilizer (log)	0.076***	0.016	-0.066***	0.020	-0.009	0.013
Climate information	-0.398***	0.050	0.411***	0.052	-0.013	0.046
CA Training	-0.186***	0.012	0.163***	0.012	0.023***	0.006
Northern	0.537***	0.081	-0.533***	0.093	-0.040	0.080
Upper West	0.200**	0.099	0.227**	0.105	0.027	0.088
Joint significance plot-level variables χ (3)	22.22***		9.41**		14.39***	
χ^2^ test of instrument	77.75					
*p* value of instrument	0.000					
No. of obs. (plots)	391		226		269	

Note: Table 3 reports the marginal effects estimates of the adoption decision from a generalized ordered probit selection model for all three adoption states (conventional tillage, short-term adoption, and long-term adoption). The *p* –value for the excluded instruments (Climate information and CA training) is reported. Significance level at

*p < 0.1

**p < 0.05

***p < 0.01

MT: Minimum tillage

In addition, we check the relevance and validity of the instruments by showing test diagnostics of a generalized method of moments (IV-GMM) estimations of the effect of the length of minimum tillage adoption on the outcomes in Table A4 in S1 Appendix. We employ the IV-GMM estimator because of its efficiency over the conventional two-stage least squares when the equation is over-identified and its robustness to heteroskedasticity [11]. The diagnostics test statistics presented in the lower part of Table A4 in S1 Appendix suggest the instruments are relevant as well as good predictors of the length of MT adoption. Specifically, the Cragg-Donald F-statistic of 23.86, the Kleibergen-Paap rk Wald F-statistic of 77.75 and the corresponding Angrist and Pischke (2009) *p*-value (*p* = 0.000) all reject the null hypothesis that the instruments are weak. Furthermore, given the Hansen J test statistic of 1.204 and the *p*-value of 0.2726, we cannot reject the null hypothesis of zero correlation between the instruments and the error term (reported in Table A4 in S1 Appendix).

## Results and discussion

### First-stage selection results

In the interest of easing interpretation, we report the marginal effects computed after estimation of the ordered probit model in columns 1, 2, and 3 of Table 3, for conventional tillage, short-term, and long-term minimum tillage adoption, respectively. In particular, the marginal effects show that male-headed households are less likely to practice conventional tillage. The results also show that an increase in extension contacts significantly decreases the probability of practicing conventional tillage by about 0.008, and increases the probability of investing in long-term minimum tillage adoption by about 0.007.

The estimates of the marginal effects also show that among credit-constrained farmers, the probability of adopting minimum tillage in the short-term increases significantly by about 0.15, while the probability of adopting minimum tillage in the long-term decreases by about 0.11. Anecdotal evidence suggests that smallholder farmers usually receive inputs free of charge from donor or government-sponsored projects within the first few years of adoption. Thus, credit constrained farmers may still be able to implement minimum tillage in the short-term. However, beyond the intervention phase, credit constrained farmers encounter liquidity constraints for the purchase of agrochemicals (e.g., herbicides, fertilizers) and may not continuously adopt minimum tillage in the long-term.

Regarding the effect of climate variability on minimum tillage adoption, we find that rainfall variability, as represented by negative rainfall shocks, significantly decrease the likelihood of farmers practicing conventional tillage by about 0.19, and increase the probability of investing in minimum tillage in the short-term by about 0.27. This finding suggests that smallholder farmers perceive the practice of minimum tillage as an adaptation strategy to mitigate the risk of unpredictable weather conditions and shortage of rainfall. This is because it has the potential to increase soil moisture by improving water infiltration and decreasing evaporation [10, 19]. The result corroborates with previous studies [e.g., 4] that found that rainfall variability influences smallholder farmers’ decisions as regards the use of soil and water conservation technologies.

We also find that increase in mean temperature increases the probability of practicing conventional tillage by 0.20 and decreases the probability of long-term adoption by about 0.17. Regarding the variables used as instruments, the results show that increase in the number of trainings on CA increases the likelihood of minimum tillage adoption in the short-term and long-term, whereas those who receive climate information are more likely to decrease the practice of conventional tillage and adopt minimum tillage in the short-term. These findings indicate that the variables used as instruments appear to be strong predictors of either adopting minimum tillage in the short-term or long-term.

### Second-stage estimation results

In this section, we present the results from the second-stage estimation in Tables 4–6 for the three adoption states, on the effect of the observed variables on maize yields, HFIAS, dietary diversity, and labor demand. To highlight the importance of self-selection, we report the correlation coefficients (*ρ*’s) in the lower parts of Tables 4–6. The estimated *ρ*’s are statistically significant across all three adoption states for maize yields and labor demand specifications. Further, the estimated correlations are statistically significant in both the short-term and long-term adoption states for HFIAS, and statistically significant only in the case of short-term adoption for the dietary diversity specification. These results indicate the presence of selection bias arising from unobserved characteristics, lending credence to the appropriateness of the ordered-probit selection model.

**Table 4:** Second stage estimates of determinants of maize yields.

Variables	Conventional tillage		Short-term MT adoption		Long-term MT adoption	
	1		2		3	
	Coefficient	S.E.	Coefficient	S.E.	Coefficient	S.E.
Age	-0.002	0.002	0.003	0.003	-0.002	0.002
Male headed	-0.042	0.043	-0.027	0.051	-0.055	0.055
HH size	0.028***	0.005	0.026***	0.009	0.019**	0.085
Education	-0.005	0.006	0.003	0.004	0.005	0.004
FBO	0.148***	0.056	-0.116**	0.057	-0.006	0.042
Farm size	0.045***	0.009	0.013	0.015	0.020**	0.009
Extension	0.046***	0.005	0.021***	0.005	0.037***	0.004
Credit access	-0.135***	0.040	-0.296***	0.056	-0.254***	0.050
Livestock	0.003	0.005	0.011***	0.004	0.008*	0.004
Mean_slope	-0.019	0.046	-0.041	0.080	0.007	0.041
Mean_fertile	-0.005	0.054	-0.146*	0.080	0.036	0.070
Mean_Mod. fertile	-0.0003	0.049	-0.192**	0.110	0.074	0.070
Asset (log)	0.033	0.024	0.095***	0.027	-0.021	0.024
Negative rainfall shock	-0.143**	0.065	-0.440***	0.115	-0.315***	0.088
Mean temperature	-0.067	0.061	0.007	0.063	0.062	0.057
Fertilizer (log)	0.168***	0.025	0.137***	0.020	0.085***	0.012
Northern	-0.060***	0.073	0.570***	0.128	0.338***	0.087
Upper West	0.204***	0.078	0.533**	0.113	0.299***	0.100
Constant	7.346******	1.700	6.379***	1.670	5.741***	1.514
*ρ*_*εμ*_	-1.109***	0.218	-0.534***	0.135	-1.027***	0.202
LR test of indep. eqs. (*ρ* = 0): χ^2^ (3) = 46.76***						
Log pseudolikelihood = -904.32						
No. of obs. (plots)	391		226		269	

Note: Columns 1, 2, and 3 present the estimates of the maize yield equation for all three adoption states, that is, conventional tillage, short-term adoption, and long-term adoption, respectively. The estimates of ***ρ***_***εμ***_ depict the correlation between the unobservables in the first-stage ordered Probit selection Eq (2) and the second-stage outcome Eqs (3 and 5) in each of the three states. SE reports the bootstrapped standard errors. Significance level at

*p < 0.1

**p < 0.05

***p < 0.01.

MT: Minimum tillage

**Table 5:** Second stage estimates of determinants of HFIAS.

Variables	Conventional tillage		Short-term MT adoption		Long-term MT adoption	
	1		2		3	
	Coefficient	S.E.	Coefficient	S.E.	Coefficient	S.E.
Age	-0.047***	0.010	-0.035***	0.010	-0.019*	0.010
Male headed	-0.504*	0.273	0.159	0.243	-0.525*	0.299
HH size	0.094**	0.037	0.035	0.037	0.146***	0.048
Education	-0.068**	0.028	-0.049	0.023	0.013	0.023
FBO	0.148	0.296	-0.691***	0.255	-0.185	0.239
Farm size	0.083*	0.049	-0.004	0.061	0.040	0.052
Extension	-0.036*	0.021	-0.014	0.022	-0.061***	0.018
Credit access	0.410*	0.245	0.323	0.252	1.021***	0.270
Livestock	0.016	0.035	-0.059**	0.024	0.028	0.028
Slope	-0.012	0.290	0.614**	0.243	-0.095	0.235
Fertile	-0.080	0.359	0.334	0.369	-0.758*	0.416
Mod. fertile	-0.078	0.346	0.168	0.362	-1.04**	0.405
Asset (log)	-0.245*	0.136	-0.123	0.118	-0.521***	0.141
Negative rainfall shock	1.863***	0.380	4.674***	0.531	1.252***	0.440
Mean temperature	0.633*	0.380	0.271	0.301	0.280	0.307
Fertilizer (log)	0.001	0.098	-0.230***	0.080	-0.070	0.058
Northern	1.029***	0.385	-0.406	0.610	-0.108	0.477
Upper West	0.284	0.521	-1.415***	0.684	-0.537	0.462
Constant	-9.603	10.230	-2.11	7.828	3.875	7.754
*ρ*_*εμ*_	-0.357	0.246	-0.333***	0.121	-0.628***	0.157
LR test of indep. eqs. (*ρ* = 0): χ^2^ (3) = 20.49***						
Log pseudolikelihood = -2403.75						
No. of obs.	207		128		154	

Note: Columns 1, 2, and 3 present the estimates of the HFIAS equation for all three adoption states, that is, conventional tillage, short-term adoption, and long-term adoption, respectively. The estimates of ***ρ***_***εμ***_ depict the correlation between the unobservables in the first-stage ordered Probit selection Eq (2) and the second-stage outcome Eqs (3 and 5) in each of the three states. SE reports the bootstrapped standard errors. Significance level at

*p < 0.1

**p < 0.05

***p < 0.01

MT: Minimum tillage

**Table 6:** Second stage estimates of determinants of dietary diversity.

Variables	Conventional tillage		Short-term MT adoption		Long-term MT adoption	
	1		2		3	
	Coefficient	S.E.	Coefficient	S.E.	Coefficient	S.E.
Age	0.001*	0.0004	0.0005	0.001	-0.001**	0.0002
Male headed	-0.002	0.011	-0.040**	0.017	0.004	0.008
HH size	0.001	0.001	-0.003	0.003	-0.002	0.001
Education	0.002	0.001	0.004***	0.001	0.002***	0.0005
FBO	0.004	0.012	0.036***	0.013	-0.009	0.007
Farm size	0.0004	0.002	0.001	0.003	0.001	0.001
Extension	0.001	0.001	0.002	0.001	-0.001	0.0006
Credit access	-0.003	0.010	-0.020*	0.013	-0.008	0.008
Livestock	0.002	0.001	0.002	0.001	-0.0003	0.001
Slope	-0.050***	0.011	-0.068***	0.015	0.004	0.006
Fertile	-0.025*	0.014	-0.007	0.033	-0.023**	0.010
Mod. fertile	0.004	0.013	-0.023	0.031	-0.008	0.009
Asset (log)	-0.006	0.006	0.042***	0.008	0.013***	0.004
Negative rainfall shock	-0.030**	0.013	-0.062**	0.027	-0.058***	0.012
Mean temperature	0.005	0.015	-0.035**	0.016	-0.019**	0.008
Fertilizer (log)	-0.006	0.004	-0.001	0.005	0.001	0.002
Northern	-0.012	0.016	-0.027	0.035	-0.017	0.015
Upper West	0.005	0.017	0.017	0.029	0.035***	0.012
Constant	0.648	0.419	-1.544***	0.402	1.405***	0.196
*ρ*_*εμ*_	0.225	0.431	0.377**	0.154	-0.189	0.369
LR test of indep. eqs. (*ρ* = 0): χ^2^ (3) = 8.04**						
Log pseudolikelihood = 484.70						
No. of obs.	207		128		154	

Note: Columns 1, 2, and 3 present the estimates of the HDDS equation for all three adoption states, that is, conventional tillage, short-term adoption, and long-term adoption, respectively. The estimates of ***ρ***_***εμ***_ depict the correlation between the unobservables in the first-stage ordered Probit selection Eq (2) and the second-stage outcome Eqs (3 and 5) in each of the three states. SE reports the bootstrapped standard errors. Significance level at

*p < 0.1

**p < 0.05

***p < 0.01

MT: Minimum tillage

The negative signs of these correlation coefficients for maize yields and HFIAS indicate positive selection on unobserved gains, suggesting that farm households with maize yields lower than that of the average farmer or food insecurity scores higher than the average farmer have higher probabilities of transitioning into short-term and long-term minimum tillage adoption. In other words, farmers decide to transition into short-term and long-term minimum tillage adoption based on their comparative advantage [51]. Also, the positive signs of the correlation coefficients related to dietary diversity (short-term minimum tillage adoption) signify reverse selection on unobserved gains, suggesting that farm households with Simpson index value above that of the average farmer have lower probabilities of transitioning into short-term minimum tillage adoption.

Next, Table 4 presents the second-stage results on the determinants of maize yields. In particular, the household size variable is positive and statistically significant across all adoption states. In other words, larger households that normally have higher labor endowments obtain higher maize yields. Farm size shows a positive and significant impact on conventional tillage, and long-term minimum tillage adoption, suggesting that among conventional tillage farmers and long-term adopters, larger farms attain higher yields compared to smaller farms. This finding contrasts the stylized fact of an inverse relationship between farm size and productivity. The results also indicate that extension contacts significantly increase maize yields across all adoption states, highlighting the crucial role extension contacts play in enhancing productivity gains. We also find that negative rainfall shock exerts a negative and statistically significant impact across all adoption states, suggesting that conventional tillage farmers, short-term and long-term minimum tillage adopters who experience rainfall stress tend to experience decreases in maize yields.

The results in Tables 5 and 6 show the determinants of HFIAS and dietary diversity for conventional tillage farmers and both short-term and long-term minimum tillage adopters, respectively.

The coefficient for household size is positive and statistically significantly different from zero in the case of HFIAS under conventional tillage and long-term minimum tillage adoption, suggesting that conventional tillage farmers and long-term adopters with larger household sizes, all things being equal, tend to be more food insecure. The variable on access to credit is positive and statistically significant in the case of HFIAS for conventional tillage farmers and long-term minimum tillage adopters, suggesting that these categories of farm households who are credit-constrained are more likely to become food insecure, partly due to their inability to purchase agriculture inputs (i.e., fertilizers, herbicides, pesticides) to increase production and farm income.

The coefficient of education is positive and statistically significant in the case of dietary diversity for short-term and long-term minimum tillage adoption. In other words, more educated farm household heads are associated with higher dietary diversity. This finding is consistent with previous studies that observed a positive and significant relationship between education and dietary quality of households [e.g. 33]. As expected, negative rainfall shock (rainfall deficit) tends to negatively affect food and nutrition security outcomes (i.e. significantly increases HFIAS and decreases dietary diversity) across all adoption states.

Similarly, a rise in mean temperature increases food insecurity (HFIAS) in the case of conventional tillage farming and decreases dietary diversity in the case of short-term and long-term adoption. Thus, climate variability exposes the crop production systems to extreme risk (e.g., through soil moisture stress, and dry spells) and has the potential to result in increased crop failure, thereby threatening the food and nutrition security of farm households [4, 37]. Table 7 also presents the output of the second-stage regressions for labor demands. In particular, the household size variable is positive and significant under both conventional tillage and short-term minimum tillage adoption, suggesting that conventional tillage farmers and short-term adopters with larger household sizes tend to have higher farm labor supply.

**Table 7:** Second stage estimates of determinants of labor demand.

Variables	Conventional tillage		Short-term MT adoption		Long-term MT adoption	
	1		2		3	
	Coefficient	S.E.	Coefficient	S.E.	Coefficient	S.E.
Age	0.004	0.003	-0.001**	0.003	0.001	0.002
Male headed	0.894	0.725	-0.023	0.082	0.059	0.075
HH size	0.0347***	0.011	0.056***	0.014	0.002	0.010
Education	0.017	0.001	0.005	0.006	0.004	0.005
FBO	0.132	0.092	-0.138	0.109	-0.030	0.055
Farm size	0.184***	0.014	0.107***	0.016	0.043***	0.009
Extension	0.017**	0.007	0.004	0.009	0.003	0.005
Credit access	-0.140**	0.066	-0.031	0.085	-0.131**	0.064
Livestock	-0.008	0.080	0.006	0.006	0.010*	0.005
Mean_fertile	-0.083	0.095	0.130	0.139	0.007	0.097
Mean_Mod. fertile	0.064	0.086	0.080	0.162	0.106	0.089
Asset (log)	0.033	0.043	0.083**	0.042	0.121***	0.027
Fertilizer (log)	0.046	0.028	0.023	0.023	-0.039***	0.013
Upper East	-0.248***	0.086	0.068**	0.111	-0.102	0.063
Upper West	0.279***	0.084	0.281***	0.083	0.056	0.064
Constant	2.614***	0.412	3.708***	0.424	3.245***	0.361
*ρ*_*εμ*_	1.032***	0.337	0.269**	0.122	0.996***	0.124
LR test of indep. eqs. (*ρ* = 0): χ^2^ (3) = 59.70***						
Log pseudolikelihood = -1239.06						
No. of obs. (plots)	391		226		269	

Note: Columns 1, 2, and 3 present the estimates of the labor demand equation for all three adoption states, that is, conventional tillage, short-term adoption, and long-term adoption, respectively. The estimates of ***ρ***_***εμ***_ depict the correlation between the unobservables in the first-stage ordered Probit selection Eq (2) and the second-stage outcome Eqs (3 and 5) in each of the three states. SE reports the bootstrapped standard errors. Significance level at

*p < 0.1

**p < 0.05

***p < 0.01

MT: Minimum tillage

### Impact of minimum tillage adoption

In this section, we disentangle the heterogeneity in the duration of adoption of minimum tillage. We present the treatment effects across transitions based on Eqs (6A) to (6D) in Table 8 for the four study outcomes discussed earlier. The estimates presented in Panel A show the treatment effects of transitioning between conventional tillage and short-term adoption, while Panel B reports the treatment effects of transitioning between short-term and long-term adoption. In particular, the ATE^†^ estimates show that transitioning from conventional tillage to short-term adoption increases maize yields, dietary diversity, and labor demand by 4.88%, 13.57%, and 8.07%, respectively, and decreases HFIAS by 13.37%. For the transition between short-term and long-term adoption, the ATE^†^ estimates show an increase in maize yields and dietary diversity by 5.41% and 24.66%, respectively, and a decrease in HFIAS and labor demand by 8.68% and 17.07%, respectively.

**Table 8:** Estimates of treatment effects parameters.

Panel A	Maize yields (log)		HFIAS		Dietary diversity		Labor demand (log)	
	1		2		3		4	
Conventional tillage vs Short-term MT adoption	Treatment effect	% of base category	Treatment effect	% of base category	Treatment effect	% of base category	Treatment effect	% of base category
ATE^†^	0.369***	4.88	-0.655***	-13.37	0.085***	13.57	0.380***	8.07
	(0.011)		(0.050)		(0.002)		(0.017)	
ATE	0.337***	4.40	-1.000***	-18.86	0.067***	10.85	0.567***	12.64
	(0.013)		(0.060)		(0.002)		(0.017)	
TT	0.271***	3.58	-0.178	-3.59	0.080***	12.84	0.573***	12.92
	(0.047)		(0.164)		(0.004)		(0.043)	
TUT	0.375***	4.88	-1.478***	-26.79	0.059***	9.68	0.563***	12.57
	(0.040)		(0.120)		(0.004)		(0.043)	
**Panel B**	**Maize yields (log)**		**HFIAS**		**Dietary diversity**		**Labor demand (log)**	
	1		2		3		4	
Short-term MT adoption vs long-term MT adoption	Treatment effect	% of base category	Treatment effect	% of base category	Treatment effect	% of base category	Treatment effect	% of base category
ATE^†^	0.430***	5.41	-0.368***	-8.68	0.175***	24.66	-0.870***	-17.07
	(0.007)		(0.071)		(0.002)		(0.012)	
ATE	0.416***	5.30	-1.229***	-27.91	0.145***	19.54	-0.703***	-13.70
	(0.010)		(0.084)		(0.003)		(0.014)	
TT	0.340***	4.33	-1.729***	-42.31	0.110***	14.22	-0.581***	-11.09
	(0.042)		(0.133)		(0.004)		(0.024)	
TUT	0.506***	6.45	-0.633***	-13.25	0.187***	26.52	-0.850***	-16.95
	(0.040)		(0.149)		(0.004)		(0.025)	

Note: This table presents the estimates of different treatment effects parameters; ATE^†^ (Average treatment effect for the full population), ATE (average treatment effect), TT (effect of treatment on the treated), TUT (effect of treatment on the untreated). Standard errors are reported in parentheses. Significance level at

*p < 0.1

**p < 0.05

***p < 0.01

As discussed in section 4, we next present the treatment effect parameters across the outcomes and their relation to the pattern of selection. More specifically, the estimates of the ATE, which measures the average effect for those at one of the transition stages, show that for a farmer chosen at random, switching from conventional tillage to short-term minimum tillage adoption increases maize yields, dietary diversity, and labor demand by 4.40%, 10.85%, and 12.64%, respectively, and reduces food insecurity (HFIAS) by 18.86%. At the same time, transitioning from short-term to long-term minimum tillage adoption increases maize yields and dietary diversity by 5.30% and 19.54%, respectively, and decreases HFIAS and labor demand by 27.91% and 13.70%, respectively.

The TT, which measures the gains for those who chose to transition, suggests that switching from conventional tillage to short-term adoption increases maize yields, dietary diversity, and labor demand by 3.58%, 12.84%, and 12.92%, respectively. At the same time, transitioning from short-term to long-term adoption increases maize yields and dietary diversity by about 4.33% and 14.22%, respectively, and decreases HFIAS and demand for labor by 42.31% and 11.09%, respectively. The TUT, which measures the gains for those who chose not to transition, suggests that switching from conventional tillage to short-term adoption increases maize yields, dietary diversity, and labor demand by 4.88%, 9.68%, and 13.57%, respectively, and reduces food insecurity by 26.79%. Whereas transitioning from short-term to long-term adoption increases maize yields and dietary diversity by 6.45% and 26.52%, respectively, it reduces food insecurity and labor demand by 13.25% and 16.95%, respectively.

The results generally exhibit substantial heterogeneity in the gains from one level of transition to the other. In particular, the magnitude of the treatment effect estimates for maize yields is relatively higher for farm households transitioning from short-term to long-term minimum tillage adoption than from conventional tillage to short-term minimum tillage adoption. These results point to a gradual increase in maize yields from minimum tillage adoption over time, favoring minimum tillage adoption in the medium- to long-term. In the context of our study, climate-related stresses such as mid-seasonal dry spells and erratic rainfall patterns, coupled with low soil quality significantly impede crop growth [56].

With its capacity to conserve soil moisture, the adoption of minimum tillage over time contributes to mitigating the effect of mid-seasonal dry spells by reducing evaporation of water, increasing crop water use efficiency, and thus stabilizing yield variability [12–14]. Further, crop residue contributes to a reduction in soil erosion, and buildup of soil organic matter over time and thus decreases production risks by increasing yields [9]. Our results corroborate other studies that showed that the benefits derived from the adoption of climate-smart agriculture practices increase with the duration of adoption [e.g., 24].

Interestingly, the results also show significant evidence of decrease in farm labor demand when transitioning into long-term minimum tillage adoption. While this observation might be surprising, it is important to underscore the fact that a substantial amount of labor is required for weeding (see *t*-test, Table 2), particularly when switching to short-term adoption, as minimum tillage systems are prone to weed infestation in the first years of implementation [14]. However, under medium to long consecutive years of adoption, there is sufficient soil surface cover, because of the continuous retention of crop residues from previous cropping seasons, which suppresses the emergence of weeds, thus contributing to a net reduction in labor requirement [14]. Consistent with our findings recent studies [*see* 12, 46] found that the adoption of conservation tillage decreases the use of labor.

Furthermore, our results reveal the pattern of selection on gains. In particular, we find evidence of reverse selection on gains (TUT>ATE>TT) between the two transitions (Panels A and B for Table 8) for maize yields, suggesting that disadvantaged farm households who are less likely to switch from conventional tillage to short-term minimum tillage adoption, and from short-term to long-term minimum tillage adoption tend to benefit more if they transition. Consistent with our analysis (see *t*-test, Table 2), conventional tillage farmers and short-term adopters are faced with a couple of limitations (such as low extension contacts) which tend to affect their yields. Thus, switching into long-term adoption helps disadvantaged farmers to significantly close the gap in maize yields. We also observe a similar pattern in the case of farm labor demand when switching from short-term to long-term minimum tillage adoption.

Our results also show evidence of positive selection on gains (TT>ATE>TUT) in the case of dietary diversity (Table 8 Panel A), suggesting that better-resourced farm households who are most likely to transition into short-term adoption of minimum tillage tend to have higher Simpson index value (i.e., higher dietary diversity). However, less endowed farm households or those who are less likely to transition into long-term minimum tillage adoption benefit the most (higher dietary diversity), rather than the least. In other words, this finding shows an equalizing effect of long-term adoption, implying a reverse selection on gains (Table 8 Panel B). We further observe a pattern of positive selection on gains in terms of HFIAS, indicating that more endowed households tend to experience better food security situations when they transition into long-term minimum tillage adoption.

**Fig 2:**
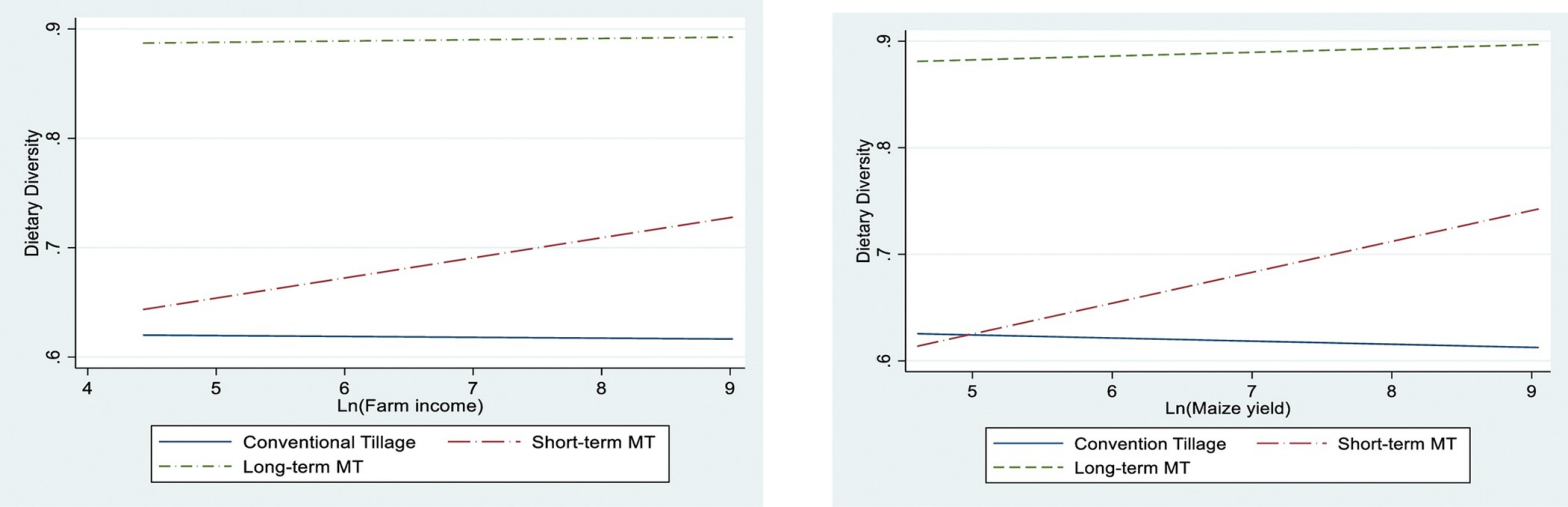
Plot of potential outcomes by household farm income and maize yields for dietary diversity.

**Fig 3:**
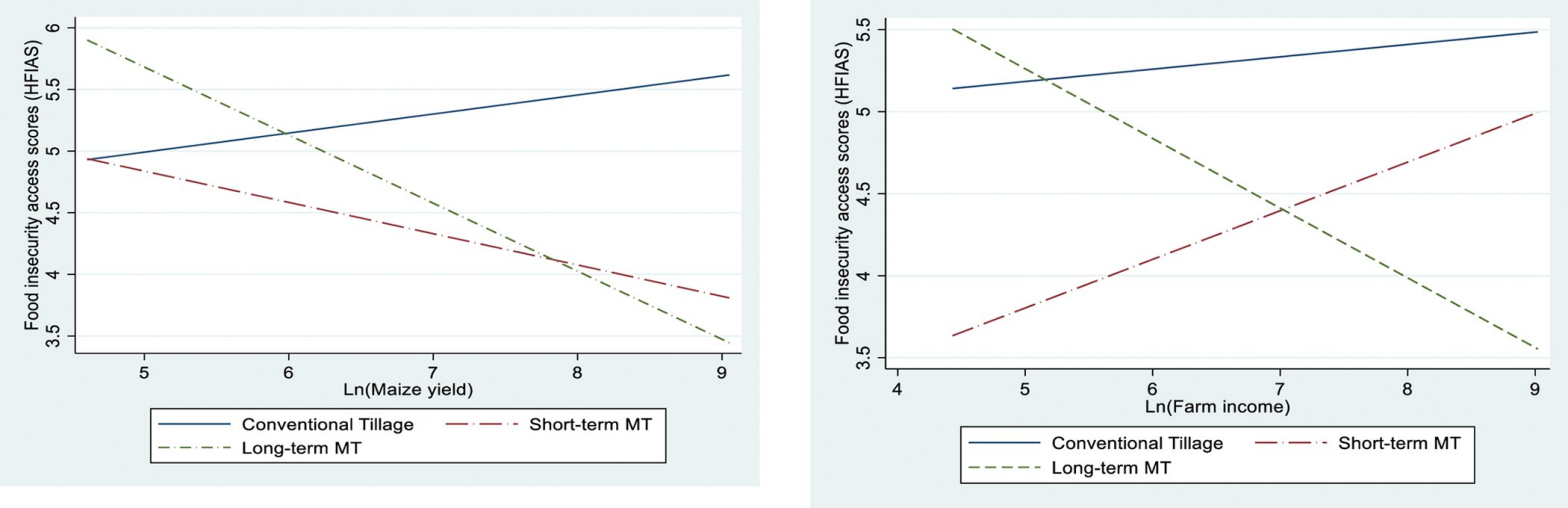
Plot of potential outcomes by household farm income and maize yields for food insecurity access scores (HFIAS).

Given the generally positive effects of minimum tillage adoption on food and nutrition security, we turn our attention to understanding the impact mechanisms. While improving food security is not a direct result of adopting minimum tillage, it can be viewed as an extension of the productivity and income-increasing effects of its adoption. A key dimension of food security is food availability which is determined by the level of food supplies. Thus, the adoption of minimum tillage is hypothesized to boost soil fertility and crop yield, thereby improving food availability. We further hypothesized that farm income contributes to better food and nutrition because it affects the overall household income, making food more accessible. In addition, during times of food scarcity and hunger, households are more likely to sell their farm produce to purchase food [8]. Figs 2 to 3 explicitly explore these relationships. Each Fig illustrates the association between household food and nutrition security for the predicted counterfactuals across the three adoption status, and maize yield and farm income. Fig 2 further shows a positive and strong association between farm income, maize yields, and dietary diversity, while Fig 3 shows a negative association between farm income and maize yields on household food insecurity access scores. This observation is further corroborated by correlation analysis in Table A4 in S1 Appendix.

## Conclusion and policy implications

This study used plot-level and household data from the northern savanna zone of Ghana and geo-referenced historical weather data to examine the determinants and impacts of adoption of minimum tillage on maize yields, household dietary diversity, and household food insecurity access scores (HFIAS), and farm labor demand. The analysis accounted for heterogeneity in the length of minimum tillage adoption (i.e., conventional tillage, short-term, and long-term minimum tillage adoptions) and estimated the treatment effects of farm households’ adoption transitions, which involved transitioning from conventional tillage to short-term adoption and from short-term to long-term adoption. We employed an ordered-probit selection model to account for potential selection bias arising from the non-randomness of minimum tillage adoption decisions. The empirical results revealed that several household and farm-level variables significantly affect farmers’ decisions regarding the use of minimum tillage. Moreover, we observed that negative rainfall shocks result in a positive and significant impact on farmers’ short-term adoption decisions, indicating farmers’ responses to rainfall variability.

Overall, our findings demonstrate that minimum tillage adoption has positive and significant impacts on maize yields and food and nutrition security across adoption transitions. In particular, and in contrast to other studies [20], we find no evidence of yield penalties often observed during the first few years of minimum tillage adoption. However, we observe relatively higher maize yields when transitioning from short-term into medium- to long-term minimum tillage adoption, potentially due to the gradual improvement in soil health (i.e., chemical, physical, and biological properties) over time. Our results also reveal a pattern of positive selection on gains from transitioning into long-term minimum tillage adoption on HFIAS, suggesting that farm households that are more likely to transition into medium- to long-term adoption tend to experience better food security situations. Also, we find that transitioning into medium- to long-term adoption results in economic benefits such as net savings in total labor demand, as the use of herbicides, and the prolonged retention of crop residue help maintain soil surface cover, thereby suppressing the emergence of weeds, and reducing the demand for labor for weed control.

A major conclusion from our results is that transitioning into longer cropping seasons under minimum tillage contributes to relatively higher maize yields and food security benefits. More importantly, differentiating between the duration of adoption is crucial in teasing out the heterogeneous benefits of minimum tillage technology. In particular, medium-to long-term investments in minimum tillage technology offers significant benefits of adapting crop production systems to climate variability. Thus, our findings highlight the importance of developing and implementing programs that not only promote adoption but also assist smallholder farmers in sustaining adoption for more cropping season. For example providing opportunities for training may improve the adoption rate of MT practice to produce a more acceptable rate of CA technology. It is important to undertake enhanced institutional and policy initiatives to remove the bottlenecks associated with the continuous adoption of minimum tillage systems. In other words, boosting extension delivery through redesigning and improving minimum tillage knowledge and information to suit local conditions may assist smallholder farmers in developing the resilience required deal with climate change.

The findings of this study should be interpreted with caution, since we primarily used cross-sectional survey data. We also accept the possibility of recall bias given that farmer recall was used to measure the length of adoption That notwithstanding, we do not expect systematic bias in our assessment. Therefore, this analysis is useful in deepening the understanding of the linkages between the length of minimum tillage adoption and food and nutrition security.

## Supporting information

S1 File (PDF)

S1 Questionnaire (DOCX)

S1 Appendix (DOCX)

S1 Data (DTA)
