# Supplementary material for: Minimum tillage as climate-smart agriculture practice and its impact on food and nutrition security
Source: PLoS One. 2023 Dec 22;18(12):e0287441. doi: 10.1371/journal.pone.0287441 (PMC10745223; doi:10.1371/journal.pone.0287441)
Supplement: S1 File — (PDF) [file pone.0287441.s001.pdf]

Christian-Albrechts University of Kiel, Germany
The Institute of Food Economics and Consumption Studies

# Contribution of Sustainable Agriculture to Household Welfare in Ghana

Thank you for participating in this interview. We are researchers from the University of Kiel, Germany. This research is being conducted as part of PhD research on sustainable agriculture. In the course of the interview, you would be asked questions about the characteristics of your farm and the household. Specifically, we are interested in learning more about the sustainable agricultural practices you carry out on your maize farms. Additionally, we would like to understand your production and sales of maize as well as any other non-farm activities of your household. Kindly note that all information provided is for research purposes only and shall be kept strictly confidential. Thank you for agreeing to participate in this interview.

| **Survey Identification** | | **General information** | |
|---|---|---|---|
| **Questionnaire number** | | **Region** | |
| **Name of enumerator** | | **District** | |
| **Code of enumerator** | | **Name of village/town** | |
| **Date of interview** | | **Language of interview** | |
| **Time started** | | **Agro-ecological zone** | 1. Sudan Savannah |
| **Village location** | | | 2. Guinea Savannah |
| **GPS coordinates of the homestead** | | | 3. Transitional zone |

**Section A: Socio-demographic characteristics of respondents**

| | |
|---|---|
| **A.1 Name of respondent** | |
| **A.2 Age** | |
| **A.3 Sex**<br>1= Male<br>2= Female | |
| **A.4 Relationship to household head**<br>1= Head 2= Spouse 3= Son/Daughter 4= Son/Daughter in law 5= Father/Mother 6= Father/Mother in law 7= Brother/Sister 8= Grandchild 9= Other relative 10= Non-relative | |
| **A.5 Marital status**<br>1= Married (monogamous) 2= Married (polygamous) 3= Single, 4= Divorced, 5= Widowed | |

| | |
|---|---|
| **A.6 Family type**<br>1= nuclear  2= extended  3= other (specify) | |
| **A.7 Household size** | |
| **A.8 Number of children** | |
| **A.9 Years of education** | |
| **A.10 Religion**<br>1= Islam  2= Christianity  3= Traditional  4= Others specify | |
| **A.11 Ethnicity/Tribe**<br>1.=Hausa  2.= Dagomba  3.= Mamprusi  4.= Gonja  5.= Grussi/Frafra  6.= Dagarti  7.= Kusasi 8.=Kassena-Nankani  9.= Konkomba  10.=Nanumba  11.= Builsa  12.= Akan  13.= Guan  14. other, specify | |
| **A.12 Status in the locality**<br>1= chief  2= village executive 3= member  4= Migrant  5= Religious leader  6= political party leader 7=other (specify) | |
| **A.13 Highest level of formal education completed (years)** | |
| **A.14 Occupation**<br>(List main and secondary occupation according to time spent)<br><br>1= Farmer (other crops, livestock and aquaculture)<br>2=Non-farm owned business (e.g. tailoring, etc); 3=Casual labourer on another farm<br>4=Civil/public servant; 5.=Housewife<br>6=Unemployed; 7= Student; 8.=Retired; 9.= Other, specify | |

| **a. Main occupation** | **b. Secondary occupation** | |
|---|---|---|
| | | |
| | | |
| | | |

| | |
|---|---|
| **A.15. Other sources of income:** Please specify | |
| **A.16. Please specify if A 14 includes option 2 (non-farm business)** | |
| **A.17 Experience in own farming activities (years)** | |
| **A.18 For how many years have you been cultivating maize?** | |
| **A.19. Are you a member of any organization/group?**<br>1= Yes 2= No | |
| **A.20. If yes, which organization/group do you belong to?**<br>1=FBO; 2=Saving and loans; 3=religious group; 4= Others specify… | |
| **Please indicate total number of household members in their respective category as in the table below** | |

| **Household member** | **Gender** | | **Age** |
|---|---|---|---|
| | Male | Female | |
| | | | |
| | | | |
| | | | |
| | | | |
| | | | |
| | | | |
| | | | |
| | | | |

**Section B. Land tenure**

We would like to ask some questions about all the land which the household used either free of charge or rented in 2018. Please record all information per plot (maximum of 5 plots).

| **Plot ID** | **Size**<br>1. Acre<br>2. Pole<br>3.Hectare | **Household member responsible for this plot**<br><br>1=Household head<br>2=Spouse<br>3=child | **Land tenure**<br><br>See code below | **Years of ownership if the farmer owns the land** | **Years of usage** | **If rented, what is the cost of renting per year (per acre)?**<br><br>(if option 6 is chosen) | **Did the household have a deed on this plot?**<br>1. Yes<br>2. No | **What kind of deed?**<br>1. Certificate (deed from Lands Commission)<br>2. Letter from village chief<br>3. Contract of sale<br>4. Other, specify | **Did the household have the right to sell the plot or to use it as collateral?**<br>1. No<br>2. Sell<br>3. Collateral<br>4. Both | **If the plot were to be sold now, how much would it be worth?** (GHS) | **Main land use**<br><br>See code below | | **What is the distance from the homestead to the plot?**<br><br>1=walking minutes<br>2=KM<br>3=Miles |
|---|---|---|---|---|---|---|---|---|---|---|---|---|---|
| | | | | | | | | | | | **a** | **b** | |
| | **LT1** | **LT2** | **LT3** | **LT4** | **LT5** | **LT6** | **LT7** | **LT8** | **LT9** | **LT10** | **LT11** | **LT12** | **LT13** |
| 1 | | | | | | | | | | | | | |
| 2 | | | | | | | | | | | | | |
| 3 | | | | | | | | | | | | | |
| 4 | | | | | | | | | | | | | |
| 5 | | | | | | | | | | | | | |

**Code LT 3**
1. Owned by respondent
2. Owned by other household member
3. Owned by family
4. Community land, distributed by the chief
5. Land made arable by the household
6. Rented-in for fixed pay
7. Sharecropper (Abunu)
8. Sharecropper (Abusa)
9. Other, specify

**Code LT11/LT12**
(*up to 2 answers per plot)*
1. Rented-out for maize cultivation
2. Rented out for cultivation of other crop production
3. Sharecropped-out for maize cultivation
4. Sharecropped-out for other crop production
7. Forest
8. Pasture
9. Bushland/wetland
10. Fallow
11. Cultivation of cash crops
12. Cultivation of maize
13. Cultivation of other food crops
14. Other, specify

## SECTION C: Plot-level Characteristics

Make sure to include all the plots owned/operated by the HH. Please record information per plot.

| Plot ID | **Slope of this plot** See code below. | **Vegetation before you started farming on the plot?** See code below | **What is the Current vegetation** See code below. | **What is the major type of soil of this plot?** 1=sandy 2=loamy 3=clay 4=laterite 5=other, specify | **What is the level of erosion of the plot** See code below | **Soil quality (fertility)** 1=infertile, 2= moderately fertile 3=fertile 4=highly fertile | **Which crops do you plant on this plot?** See code below | **What was the use of this plot prior to planting maize?** See code below | | **Were there trees on the plot before?** (1=yes, 2=no) | **If yes what type of trees?** 1=Cashew 2=Timber 3=Dawadawa 4=Mango 5=Cocoa 6=Timber 7=others….. | **Have you cut down some of the trees since you started farming on the plot?** (1=yes, 2=no) |
|---|---|---|---|---|---|---|---|---|---|---|---|---|
| | | | | | | | | 2018 farming season | 2017 farming season | | | |
| | **PL1** | **PL 2** | **PL 3** | **PL 4** | **PL 5** | **PL 6** | **PL 7** | **PL 9** | **PL 10** | **PL 11** | **PL 12** | **PL 13** |
| 1 | | | | | | | | | | | | |
| 2 | | | | | | | | | | | | |
| 3 | | | | | | | | | | | | |
| 4 | | | | | | | | | | | | |
| 5 | | | | | | | | | | | | |

**Code for PL 1**
1=flat
2=moderate
3=steep

**Code for PL 3**
1=forest,
2= semi forest,
3= savanna

**Code for PL 9-10**
1=rented-out
2=fallow
3=tree plantation
4=virgin land
5=grazing
6=maize
7=rice
8=cereals other than maize/rice
9=legumes10=root/tuber
11=vegetables 12=fruits
13=banana 14=flower
15=other crop (specify)
16=borrowed-out

**Code for PL 2**
1=forest (>200 trees),
2=semi-forest (200 to 50 tree)
3=savanna (<50 trees)

**Code for PL 5**
1=no erosion
2=minimal erosion
3=moderate erosion
4=eroded
5=severe erosion)

**Code for PL 7**
1. Maize
2. Cashew
3. Cotton
4. Groundnut
5. Sorghum
6: Mango
7. Millet
8. Rice
9. Soybeans
10. Yam
11. Vegetables
12.Other, specify

## Section D: Adoption of Sustainable Agricultural Strategies (SASs)

This section intends to know your current usage of sustainable agricultural strategies. These questions should be repeated for each of the farmer's.

| | Have you ever used this technology for your maize production?<br><br>If yes, in which year did you use it for the first time?<br><br>If no, why have you never adopted this technology? | | | | Did you discontinue the use of SASs at any point?<br><br>If yes, in which year did you discontinue? If there are several times of discontinuation, please answer about the last one.<br><br>If yes, why did you discontinue this SASs? | | | Did you re-adopt the SAS?<br><br>If yes, in when (year) did you re-adopt?<br><br>If there are several times of re-adoption, please answer about the last one after the last discontinuation. | | How did you acquire knowledge on SASs? **(Code SA10)** |
|---|---|---|---|---|---|---|---|---|---|---|
| **Sustainable Agricultural Strategies (SASs)** | 1=yes<br>2=no | If 1=yes, in which year? | Extent of adoption<br><br>*(in terms of plot size)* | If 2=no, reason for non-adoption.<br><br>See code below | 1=yes<br>2=no | If 1=yes, in which year? | If 1=yes, reason for discontinuation. See code below | 1=yes<br>2=no | If 1=yes, in which year? | |
| | **SA1** | **SA2** | **SA3** | **SA4** | **SA5** | **SA6** | **SA7** | **SA8** | **SA9** | **SA10** |
| **Conservation Agriculture** | | | | | | | | | | |
| Minimum/zero tillage | | | | | | | | | | |
| Cover cropping | | | | | | | | | | |
| Mulching | | | | | | | | | | |
| Use of leguminous crops | | | | | | | | | | |
| Crop rotation | | | | | | | | | | |
| Intercropping | | | | | | | | | | |
| Ripping | | | | | | | | | | |
| Others, specify………………… | | | | | | | | | | |
| **Crop diversification** | | | | | | | | | | |
| Using improved /drought resistant varieties | | | | | | | | | | |
| Changing planting date | | | | | | | | | | |
| Changing crops | | | | | | | | | | |

| | | | | | | | | | | |
|---|---|---|---|---|---|---|---|---|---|---|
| Other, specify……………… | | | | | | | | | | |
| **Soil and Water Conservation** | | | | | | | | | | |
| Grass stripping | | | | | | | | | | |
| Contour ploughing | | | | | | | | | | |
| Terracing | | | | | | | | | | |
| Stone bunding | | | | | | | | | | |
| Earth bunding | | | | | | | | | | |
| Zai technique | | | | | | | | | | |
| Others, specify……………………………. | | | | | | | | | | |
| **Soil Fertility** | | | | | | | | | | |
| Application of manure | | | | | | | | | | |
| Compost Application | | | | | | | | | | |
| Organic fertilizer | | | | | | | | | | |
| Others, specify……………………………. | | | | | | | | | | |

**Code for SA4/SA7: 1**=not aware of this technology **2**=lack of technical know-how **3**=lack of cash/credit 4=not profitable. **5**=unavailability of labor **6**=unavailability of product (herbicide/fertilizer/improved variety seed) **7=**unavailability of service (tractor) **8**=others, specify.

**Code for SA10**: **1**=own experimentation **2**=observation of neighboring farmers (without direct instructions) **3**=taught by neighboring farmers **4**=government extension agents **5**=media **6**=non-government agencies **7**=private institutions **8**=others, specify

| **Sustainable Agricultural Strategies (SASs)** | **SA12. Why do you practice these strategies?** *You can select more than one reasons*. | | | | |
|---|---|---|---|---|---|
| | **SA12a** | **SA12b** | **SA12c** | **SA12d** | **Codes SA12** |
| **Conservation Agriculture** | | | | | **1**=Improve production<br>2=Reduce, prevent, restore land degradation (soil, water, vegetation)<br>3=Conserve ecosystem<br>4=protect a watershed/ downstream areas – in combination with other Technologies<br>5=Preserve/ improve biodiversity<br><br>6=Reduce risk of disasters (e.g. droughts, floods, landslides)<br>7=Adapt to climate change/ extremes and its impacts (e.g. resilience to droughts, storms)<br>8=Mitigate climate change and its impacts (e.g. through carbon sequestration)<br>9=Create beneficial economic impact (e.g. increase income/ employment opportunities)<br>10=create beneficial social impact (e.g. reduce conflicts on natural resources, support marginalized groups)<br>11=other purpose (specify)……………………………… |
| Minimum/zero tillage | | | | | |
| Cover cropping | | | | | |
| Mulching | | | | | |
| Use of leguminous crops | | | | | |
| Crop rotation | | | | | |
| Intercropping | | | | | |
| Ripping | | | | | |
| Others, specify………………… | | | | | |
| **Crop diversification** | | | | | |
| Using improved /drought resistant varieties | | | | | |
| Changing planting date | | | | | |
| Changing crops | | | | | |
| Other, specify……………… | | | | | |
| **Soil and Water Conservation** | | | | | |
| Grass stripping | | | | | |
| Contour ploughing | | | | | |
| Terracing | | | | | |
| Stone bunding | | | | | |
| Earth bunding | | | | | |
| Zai technique | | | | | |
| Others, specify……………………………. | | | | | |
| **Soil Fertility** | | | | | |
| Application of manure | | | | | |
| Compost Application | | | | | |
| Organic fertilizer | | | | | |
| Others,specify……………………………. | | | | | |

**SA13. Have you ever increased your farm size in the last two farming seasons?** 1=yes; 2=no

**SA14. If yes, why did you increase your farm size?**......................................................................

**SA15. If SA13 is yes, by how many acres have you increased your farm land?** …………………….

**SA16. If SA13 is no, why haven't you increased your farm size**?................................................................

**Section E. Perception of sustainable agricultural strategies**

First, tick relevant impacts (tick boxes on the left, several answers possible). Then, for each selected impact, tick the extent and specify/ quantify if possible.

**P1. Socio economic impacts**

| | | 1 | 2 | 3 | 4 | 5 | 6 | 7 | |
|---|---|---|---|---|---|---|---|---|---|
| **Production** | decreased | | | | | | | | increased |
| crop production | decreased | | | | | | | | increased |
| crop quality | decreased | | | | | | | | increased |
| production area (new land) | decreased | | | | | | | | increased |
| **Income and costs** | decreased | | | | | | | | increased |
| expenses on agricultural inputs | decreased | | | | | | | | increased |
| farm income | decreased | | | | | | | | increased |
| diversity of income sources | decreased | | | | | | | | Increased |
| **Other socioeconomic impacts** | | | | | | | | | |
| | | | | | | | | | |

**P2. Sociocultural impacts**

| | | 1 | 2 | 3 | 4 | 5 | 6 | 7 | |
|---|---|---|---|---|---|---|---|---|---|
| food security/ self-sufficiency | decreased | | | | | | | | increased |
| health situation | worsened | | | | | | | | improved |
| Network among members | worsened | | | | | | | | improved |
| Migration | decreased | | | | | | | | improved |
| **Other sociocultural impacts** | | | | | | | | | |
| | | | | | | | | | |

**P3. Ecological impacts**

| | | 1 | 2 | 3 | 4 | 5 | 6 | 7 | |
|---|---|---|---|---|---|---|---|---|---|
| **Soil** | decreased | | | | | | | | increased |
| soil moisture | decreased | | | | | | | | increased |
| soil organic matter | decreased | | | | | | | | increased |
| soil loss | increased | | | | | | | | decreased |
| **Biodiversity: vegetation,** | | | | | | | | | |
| vegetation cover | decreased | | | | | | | | increased |
| pests/ diseases | decreased | | | | | | | | increased |
| **External inputs** | | | | | | | | | |
| Inorganic fertilizer | increased | | | | | | | | decreased |
| Pesticides | increased | | | | | | | | decreased |
| Herbicides | increased | | | | | | | | decreased |

**1**=Very negative (– 50-100%); **2**=Negative (– 20-50%) **3**=slightly negative (– 5-20%); **4**=Negligible impact; **5**=slightly positive (+5-20%); **6**=Positive (+20-50%); **7**=Very positive (+50-100%)

## SECTION E. Maize crop budget

### E.1. Input use for Maize Production.

The questions below refer to 2018 farming season**.** Please name all external inputs (inorganic fertilizer, herbicides, pesticides) applied on the maize plots between January and December 2018. ***Please refer to Section C***.

| | **Planting** | | | | | | | **Inorganic fertilizer Use** | | | | | | **Herbicide** | | | | **Other Chemical** | |
|---|---|---|---|---|---|---|---|---|---|---|---|---|---|---|---|---|---|---|---|
| | | | | | | | | | **If yes to MP08** | | | | | | | | | | |
| | | | | | | | | | 1st application | | 2nd application | | | | | | | | |
| **Plot ID** | What was the area planted to maize? (acre) | Name of the Maize Variety See code below | How did you obtain the seed? See code below | From whom did you obtain the seed? See code below | Quantity of seed used on this plot (kg) | Total Cost of seed (kg) used in GHC | How did you sow this crop on this plot in farming season 2018? See code below | Did you apply fertilizer? 1=Yes 2=No | Type of fertilizer See code below | Quantity used (kg) | Type of fertilizer See code below | Quantity used (kg) | **If MP08=1** Total cost of fertilizer | Did you use herbicide for weeding during this crop growing stage? 1=Yes 2=No | **If MP14 =1** what is the quantity used? | **If MP14=1** Total cost of herbicide used (GHC) | Did you use insecticide /fungicide? 1=Yes 2=No | **If MP17=1,** what is the quantity used? | **If MP16=1** Total cost of other chemicals (GHC) |
| | **MP01** | **MP02** | **MP03** | **MP04** | **MP05** | **MP06** | **MP07** | **MP08** | **MP09** | **MP10** | **MP11** | **MP12** | **MP13** | **MP14** | **MP15** | **MP16** | **MP17** | **MP18** | **MP19** |
| 1 | | | | | | | | | | | | | | | | | | | |
| 2 | | | | | | | | | | | | | | | | | | | |
| 3 | | | | | | | | | | | | | | | | | | | |
| 4 | | | | | | | | | | | | | | | | | | | |
| 5 | | | | | | | | | | | | | | | | | | | |

**Codes for MP02**
1=Obatanpa;
2=Golden Jubilee;
3=Mamaba (hybrid);
4=Abelechi (Abeleehi);
5=Dorke SR;
6= Okomasa;
7=Etubi (hybrid);
8=Dodzi;
9=Omankwa;
10=Akposoe;
11=Abontem;
12=Golden Crystal;
13=Sanzal sima
14=Ewul buoyo
15=Wang data
16=Agric;
17=local/traditional/;
18= do not know;
19=imported hybrid (specify _________);
20=Other(specify_______

**Code for MP03**
1=Purchased without subsidy
2=In-kind credit
3=Gifted 4=Own production;
5=Purchased through subsidy

**Code for MP04**
1=Extension worker
2=Other farmer in the village
3=Other farmer outside the village
4=Own production
5=Local market
6=Trader
7=Other(specify)

**Code for MP07**
1=broadcasting before ploughing
2=broadcasting after ploughing
3=dibbling randomly
4=dibbling in a line
5=drilling
6=transplanting (exclude cases
Where transplantation was used for only small part of the plot)

**Code for MP09/MP11**
1=NPK
2=Urea
3=ammonium sulfate
4=other, specify

**E.2. Input costs – hired and family labor**

**L1.** What was the wage rate per man-day during 2018 farming season?…………………………………………..

**L2.** Was the male wage rate different from that female workers? 1=Yes 2=No

**L3.** If yes, what was the rate for female?..........................................................

**L4.**Please count all hired and family laborers during the 2018 farming on the farmer's maize plots. Please count working days for each task for each plot for the whole year 2018. Please include cash and in-kind payments when calculating the average pay per day by task. Please repeat these questions for each plot if possible.

| **Plot ID** | **Activity** | **L5. Did you hire any worker?** 1.Yes 2.No | **L6. Type** 1=Permanent 2=temporary) | **L7. Number of hired workers** | | | **L8. Total number of man days** | **L9. Did you use family labour?** 1.Yes 2.No | **L10. Number of family labour** | | | **L11. Total number of man days per each worker** | **L12. Cost of family per man-day.** |
|---|---|---|---|---|---|---|---|---|---|---|---|---|---|
| | | | | **L7a. male** | **L7b. female** | **L7c Total no. of labourers** | | | **L10a. Adult Men** | **L10b. Adult Female** | **L10c.Children Under 15 yrs.** | | |
| | Land preparation and ploughing or ripping | | | | | | | | | | | | |
| | Harrowing | | | | | | | | | | | | |
| | Planting | | | | | | | | | | | | |
| | **Applying agrochemical inputs** | | | | | | | | | | | | |
| | Fertilizer | | | | | | | | | | | | |
| | Insecticide/pesticide | | | | | | | | | | | | |
| | Herbicide/weedicide | | | | | | | | | | | | |
| | **Weeding control** | | | | | | | | | | | | |
| | 1st weeding | | | | | | | | | | | | |
| | 2nd weeding | | | | | | | | | | | | |
| | 3rd weeding | | | | | | | | | | | | |
| | **Sustainable agricultural strategies** | | | | | | | | | | | | |
| | Harvesting | | | | | | | | | | | | |
| | Shelling | | | | | | | | | | | | |
| | Bagging and weighing | | | | | | | | | | | | |
| | Storing | | | | | | | | | | | | |

**E.3. Maize input costs – other expenditures**

Please list all other expenditures related to maize cultivation on the farmer's plot incurred during 2018 farming season.

| | | **Total amount spent in 2018** (GHS) |
|---|---|---|
| **OE1** | Cost of implementing sustainable agricultural strategies | |
| **OE2** | Purchase or rental of agricultural equipment (e.g. spaying machines, hand tools, bags, drying mats) | |
| **OE3** | Transportation costs (e.g. for buying inputs, bringing maize from farm to home, bringing maize to point of sale) | |
| **OE4** | Other farm-related expenditures, specify | |

## Section F: Use of external inputs

| | External input use | Herbicide | Fertilizer | Pesticide | Others |
|---|---|---|---|---|---|
| EI1 | In which year did you start using external input (s)? | | | | |
| EI2 | Have you been applying external inputs(s) on your farm in the last 3 farming seasons? 1=Yes 2=No | | | | |
| EI3 | If yes, why are you using it?................................................. | | | | |
| EI4 | If no, why are you no using it?.............................................. | | | | |
| EI5 | Name of external input(s) you are using currently | | | | |
| EI6 | Have you reduced the amount of external inputs use? 1=Yes 2=No | | | | |
| EI7 | If yes, by how much? Please quantity in percentage terms. | | | | |
| EI8 | What is your recommended dosage (lit./Kg /acre**)** | | | | |
| EI9 | Were you using another one before 1=yes, 2=No | | | | |
| E20 | If yes, why did you stop using it? | | | | |
| E21 | Who recommended to you what you are currently using? | | | | |
| E22 | Who applies it for you on your farm? | | | | |
| E23 | If self, are you trained on the use 1=yes; 2=No | | | | |

## Section G. Maize Harvest

Please indicate the quantity of maize harvested with the corresponding plot ID, as well as the distribution of the harvests during 2018 farming season.

| Plot ID | Quantity of maize harvested (kg) | Quantity consumed (kg) | Quantity sold (kg) | Quantity stored as seed (kg) |
|---|---|---|---|---|
| | MH1 | MH2 | MH3 | MH4 |
| 1 | | | | |
| 2 | | | | |
| 3 | | | | |
| 4 | | | | |
| 5 | | | | |

## Section H. Maize sales

**MS1. How do you access market information?** 1=neighbor, 2=FBO, 3=Mobile phone, 4=Radio, 5=Extension agents, 6=others specify…

Please ask for all maize sales of the respondent for the produce harvested in 2018 farming season. One maize sale includes all the maize sold to the same buyer at the same price. In case you sell to the same buyer later for a different price, it is counted as another sale. Please ask these questions for each plot.

| Sale ID | MS2. Maize revenue | | | To whom did you sell?<br>1. Farmer; 2. local trader<br>3. Institution/company<br>4. Other, specify | Where did you sell the produce?<br>1-Farm gate<br>2-Local village<br>3-Neighboring town/village<br>4-big town market 5. Other, specify | When did you sell?<br>[month, aggregate several sales to one buyer within one month] | Do you have a written /verbal contract with your buyer?<br>1= yes, 2=no | What is the distance to nearest market?<br>1=walking distance<br>2=KM; 3=Miles;<br>4=Others specify |
|---|---|---|---|---|---|---|---|---|
| | **Quantity sold** (refer to Section G, MH3) | **Unit** | **Price per unit** (GHS) | | | | | |
| | MS2a | MS2b | MS2c | MS3 | MS4 | MS5 | MS6 | MS7 |
| 1 | | | | | | | | |
| 2 | | | | | | | | |
| 3 | | | | | | | | |
| 4 | | | | | | | | |
| 5 | | | | | | | | |

**SECTION I. Other crop production**

**OC1**. Do you cultivate other crops apart from maize? 1=Yes 2=No

If yes, Please complete the table below.

The following questions refer to other crops which the household cultivated during the 2018 farming season on all plots that the household owns or has access to. This also includes crops that are intercropped with maize on the respondent's plots. Please only record crops (from 2018 harvest) that are sold and not only grown for home consumption. You can list up to 3 crops per plot.

| **Plot ID** | **Crop type** | | | **What is the area (in the specified unit) planted to these crops?**<br><br>**Unit**<br>1. Acres<br>2. Plot<br>3. Pole | | | **What is the main purpose of cultivating this crop?**<br><br>1.Home Consumption<br>2. For the market | | | **Total yield** (please specify the unit)<br>See code below | | | **How much was sold? (kg)** | | | **Price per unit (kg)** (GHS) | | | **What is total cost of planting seeds** (GHS) | | | **What is the total expenditure for agrochemical inputs (fertilizer, Insecticides)?** | | | **Please state other expenditures if any** | | |
|---|---|---|---|---|---|---|---|---|---|---|---|---|---|---|---|---|---|---|---|---|---|---|---|---|---|---|---|
| | **OC2** | **OC3** | **OC4** | **OC5** | **OC6** | **OC7** | **OC8** | **OC9** | **OC10** | **OC11** | **OC12** | **OC13** | **OC14** | **OC15** | **OC16** | **OC17** | **OC18** | **OC19** | **OC20** | **OC21** | **OC22** | **OC23** | **OC24** | **OC25** | **OC26** | **OC27** | **OC28** |
| **1** | **a** | **b** | **c** | **a** | **b** | **c** | **a** | **b** | **c** | **a** | **b** | **c** | **a** | | **b** | **c** | | **a** | **b** | **c** | **a** | **b** | **c** | **a** | **b** | **c** | |
| **2** | | | | | | | | | | | | | | | | | | | | | | | | | | | |
| **3** | | | | | | | | | | | | | | | | | | | | | | | | | | | |
| **4** | | | | | | | | | | | | | | | | | | | | | | | | | | | |
| **5** | | | | | | | | | | | | | | | | | | | | | | | | | | | |

**Code OC2-OC4**
1. Cashew
2. Cotton
3. Groundnut
4. Sorghum
5: Mango
6. Millet
7. Rice
8. Soybeans
9. Yam
10. Other, specify

**Code OC11-13**
1. Bowl
2. Hundred
3. Kg
4. Maxi bag
5. Metric ton
6. Mini bag
7. Pound
8. Other, specify

**SECTION J: Migration**

| | |
|---|---|
| **M1. Were you born in this village/community?**<br>1. Yes ; 2. No | |
| **M2. If no, when did you move to this village/community?** (year) | |
| **M3. Place of birth**<br>1=Outside this village, same district; 4=Outside Ghana<br>2=Outside this district, same region;<br>3=Other region in Ghana | |
| **M4. Why did you move to this village/community instead of somewhere else?**<br>[PLEASE LIST ORDER OF IMPORTANCE]<br><br>1 High production and productivity of agriculture 2.Availability of fertile lands /high soil fertility 3. High level of income 4. Employment, seasonal employment 5. Small sized land holdings 6. Good educational facilities 7. Adequate health-care services 8. Lack of infrastructural facilities 9. Evil social customs and practices 10. Others. | |
| **M5. Have you ever lived outside this area [DISTRICT/TOWN/CITY] in the last 3 years?**<br>1. Yes ; 2. No | |
| **M6. If yes, where did you go?**<br><br>1=Outside this village, same district 4=Burkina Faso 7=Nigeria<br>2=Outside this district, same region 5=Côte d'Ivoire 8=Togo<br>3=Other region in Ghana 6=Mali 9=Other, specify | |
| **M7. If yes, for how long?** | |
| | |
| **M8. Has any household member ever lived outside this area** [DISTRICT/TOWN/CITY] in the last 3 years?<br>1=Yes; 2=No | |
| **M9. If yes, how many?** | |
| **M10. How long did they stay?** | |
| **M11. What were the reasons for migrating?**<br><br>[PLEASE LIST ORDER OF IMPORTANCE]<br><br>1 Low production and productivity of agriculture 2.Scarcity of fertile lands /low soil fertility 3. Low level of income4. Unemployment, under-employment, seasonal unemployment 5. Small sized land holdings 6. Act of proper educational facilities 7. Lack of health-care services 8. Lack of infrastructural facilities 9. Evil social customs and practices 10. Others. | |
| **M12. Do you are any member of your household plan migrating to any other town/city?**<br>1=Yes; 2=No | |
| **M13. If yes, how long?** | |
| **M14. If yes, what is the reason for the planned migration?**<br>1=Access to fertile land; 2=off farm labour; 3=education; 4=others specify…………….. | |

## SECTION K: Household Assets

| Asset | | A1. Number of items solely by owned HH | A2. Total value (GHC) (current value) | In the past 12 months | | Asset | | A1. Number of items solely owned by HH | A2. Total value (GHC) (current value) | In the past 12 months | |
|---|---|---|---|---|---|---|---|---|---|---|---|
| | | | | A3a. Number of items **purchased** | A3b. Number of items **sold** | | | | | A3a. Number of items **purchased** | A3b. Number of items **sold** |
| **Farm implement** | | | | | | **Other Items** | | | | | |
| Tractor | 1 | | | | | Bicycle | 14 | | | | |
| Plough | 2 | | | | | Radio | 15 | | | | |
| Carts | 3 | | | | | Iron | 16 | | | | |
| Wheelbarrows | 4 | | | | | TV | 17 | | | | |
| Harrow | 5 | | | | | Mobile Phone | 18 | | | | |
| Motorised spraying machine | 6 | | | | | Satellite dish/ Multi TV/DSTV | 19 | | | | |
| Knapsack sprayer | 7 | | | | | Chair/Sofa | 20 | | | | |
| Water tanks | 8 | | | | | Table | 21 | | | | |
| Beehives | 9 | | | | | Bed/ matrasses | 22 | | | | |
| Trailers | 10 | | | | | Refrigerator | 23 | | | | |
| Grinders | 11 | | | | | Motorbike | 24 | | | | |
| Hand hoe | 12 | | | | | Vehicle | 25 | | | | |
| Husking machine | 13 | | | | | Tricycle | 26 | | | | |
| Other agricultural equipment, specify | 50 | | | | | | | | | | |

**On the respondent's house**

**A4:** Is this house your own? **1= yes 2=no**

**A5:** How old is this house ?..........................................................years old

**A6:** What is the worth of your house now?....................................GHC (approximation)

**A7:** Roof material (multiple response applicable) 1=grass thatched; 2= iron sheet; 3=roofing tile; 4=wood; 5=cement/concrete; 6=other……………………..

**A8:** Wall material?...........................................1=mud; 2=bricks/stones; 3=wood; 4=iron sheet; 5=other…………………………………

**A9:** Floor Material?................................................1=cement; 2=earth; 3=other…………………………

**SECTION L: Livestock**

| Livestock Type | Livestock Code | Oct 2017 | | | Sept 2018 | | | Change in Number in the last 12 months | | |
|---|---|---|---|---|---|---|---|---|---|---|
| | | Number Owned by HH members | | Total value in GHC | Number Owned by HH members | | Total value in GHC | Number Consumed at home | Number bought | Number sold |
| **NAME** | **LI CODE** | **LI1a. Male** | **LI1b. Female** | **LI2** | **LI3a. Male** | **LI3b. Female** | **LI4** | **LI5** | **LI6** | **LI7** |
| Cows | 1 | | | | | | | | | |
| Goats | 2 | | | | | | | | | |
| Sheep | 3 | | | | | | | | | |
| Chicken | 4 | | | | | | | | | |
| Pigs | 5 | | | | | | | | | |
| Donkeys | 6 | | | | | | | | | |
| Guinea Fowls | 7 | | | | | | | | | |

## SECTION M: FOOD SECURITY

FS0. Was the Household able to provide adequate food for its members during the year 2018? **0=No 1=Yes**

FS1. Which months of the year does the household have food in abundance (multiple choice) 1=Jan 2=Feb 3=Mar 4=Apr 5=May 6=Jun 7=Jul 8=Aug 9=Sept 10=Oct 11=Nov 12=Dec

FS2. Which months of the year does the household have just adequate food? (Multiple choice) 1=Jan 2=Feb 3=Mar 4=Apr 5=May 6=Jun 7=Jul 8=Aug 9=Sept 10=Oct 11=Nov 12=Dec

FS3. Which months of the year does the household barely have enough food (lean period) (multiple choice) 1=Jan 2=Feb 3=Mar 4=Apr 5=May 6=Jun 7=Jul 8=Aug 9=Sept 10=Oct 11=Nov 12=Dec

FS4: Did you or any household member go hungry within a whole day and night without eating anything because there was not enough food? 1=yes, 2=no

FS5: If yes, how often did it occur in 2018?

If No, how did you manage food distribution in the household?

| | Abundance period | Period of average availability | Lean period |
|---|---|---|---|
| Number of meals per day (on average) | | | |
| If less than 3, give reasons | | | |
| Number of days per month you took only two meals per day because of lack of money/food | | | |
| Number of days per month you took only one meal per day because of lack of money/food | | | |
| Number of days without food in the period because of lack of money/food | | | |
| Number of months in which you did not have enough food to meet your family's needs (cut the size of your meals)? | | | |

**FS 6: Dishes eaten and ingredients for each dish by period**

| Periods | Major food ingredient | | | | | |
|---|---|---|---|---|---|---|
| | Food groups for HDDS | Code | No. days consumed within a week | Quantity per day | **Code for food groups** | **Unit Code** |
| Abundance period | Cereals, roots, and tubers | | | | 1=rice, 2=maize, 3=millet, 4=sorghum, 5=cassava, 6=groundnut, , 7=cowpea, 8=onion, 9=tomato, 10=sweet potato, 11= eggplant, 12=okra, 13=banana, 14=mango, 15=Pineapple, 16=orange, 17=cashew nut, 18=potatoes, 19=yam, 20=bambara beans, 21=beans, 22=leafy vegetables, 23=peanuts, 24=okra, 25=palm nut, 26=cowpea (leaves), 27=fish and seafood, 28=meat , poultry, guinea fowl, 29=snail, 30=egg, 31=oil/fats, 32=milk and milk products, 33=lettuce, 34=Other specify | 1=90 kg bag<br>2=50 kg bag<br>3=25 kg bag<br>4=10 kg bag<br>5=2 kg bag<br>6=kgs<br>7=grams<br>8=litres<br>9=tones<br>10=numbers<br>11=bunch (banana)<br>12=wheelbarrow<br>13=cart load<br>14=crate<br>15=head load<br>16=other (specify**)** |
| | Pulses and legumes | | | | | |
| | Vegetables | | | | | |
| | Fruits | | | | | |
| | Meats, fish and seafood, and eggs | | | | | |
| | Dairy products | | | | | |
| | Oils and fats | | | | | |
| Period of average availability | Cereals, roots, and tubers | | | | | |
| | Pulses and legumes | | | | | |
| | Vegetables | | | | | |
| | Fruits | | | | | |
| | Meats, fish and seafood, and eggs | | | | | |
| | Dairy products | | | | | |
| | Oils and fats | | | | | |
| Lean period | Cereals, roots, and tubers | | | | | |
| | Pulses and legumes | | | | | |
| | Vegetables | | | | | |
| | Fruits | | | | | |
| | Meats, fish and seafood, and eggs | | | | | |
| | Dairy products | | | | | |
| | Oils and fats | | | | | |

**Section N: Expenditure on Food and non-food items**

Indicate how much the household spend on food and non-food items (listed in section M) in the last month.

| Item | Amount | Item | Amount |
|---|---|---|---|
| **Food expenditure** | | **Non-food expenditure** | |
| Cereals, roots, and tubers | | Salt | |
| Pulses and legumes | | Coffee/Tea: powder | |
| Vegetables | | Drinks (including alcohol) | |
| Fruits | | Tobacco/Cigarettes | |
| Meats, fish and seafood, and eggs | | Electricity | |
| Dairy products | | Communication | |
| Oils and fats | | Firewood | |
| Salt | | Cow dung | |
| Coffee/Tea: powder | | Charcoal | |
| Drinks (including alcohol) | | Kerosene/Gas | |
| | | Soap/washing products | |
| | | School fee, textbooks, etc | |
| | | Medical care | |
| | | Transportation | |
| | | Clothing/Shoes | |

**SECTION O: Extension and Training**

| HH See code below | Did any member of HH received any extension visits in the last year? 1=yes 2=no | Extension service provider See code below | Frequency of visit See code below | How satisfied are you with the content of information provided by the extension service? | Did any member receive training 1=yes 2=no | Provider of training See Code below | What kind of training? | Number of days of training in the last season | How satisfied are you with the content of training provided by the extension service? | What is the distance to the extension office from homestead? 1=walking minutes 2=KM 3=Miles |
|---|---|---|---|---|---|---|---|---|---|---|
| | **EX1** | **EX2** | **EX3** | **EX4** | **EX5** | **EX6** | **EX7** | **EX8** | **EX9** | **EX10** |
| | | | | | | | | | | |
| | | | | | | | | | | |
| | | | | | | | | | | |

**Code for EX2/EX6**: 1=agricultural extension agents, 2=extension worker from NGO, 3= private extension agents, 4=Farmer based organization, 5=other (specify)

**Code for EX3:** 1=weekly, 2=monthly, 3=once in 3 months, 4=once every 6 months, 5= Once a year, 5=others, specify……………………..

**HH**: 1. Household head 2. Spouse 3.Children (Male adults) 4.Children (Female adults);

**Code for EX7:** 1=Sustainable Agricultural Strategies, 2=Maize production; Farming as a business, 3= Records Keeping, 4=others, please specify………… **Code for EX4/EX9:** 1=very satisfied, 2=somewhat satisfied, 3=not satisfied, 4=totally unsatisfied

**SECTION P: Access to credit**

AC1. Do you face credit constraints in your farm operations? 1=yes; 2=no

AC2: If yes, do you source credit to finance your farm operations?............. 1=yes; 2=no

AC3: If no, please state the reasons for not sourcing credit? ..................................................................................

AC4: If yes, please indicate name of credit agency...................................................................................................

AC5: What are your other sources of finance for your farm operations?.................................................................

AC6: Did you source credit for your farm business in the 2018 farming season? -------- 1=yes; 2=no

AC7: If yes, what form was it ................................. 1=cash; 2= in-kind (3) Other (specify)…………………………

AC8: Did you pay with interest?............................ 1=yes; 2=no

AC9: What was the interest rate?................................ (in percentage)

AC10: what was the average repayment period ...............................months?

AC11: Are you aware of other farming credit sources in your locality? .............. 1=yes; 2=no

AC12: If yes please name them...................................................................................................................................

AC13: Have you sourced credit from them before?.........1=Yes; 2=No

AC14: If yes, do you still source credit from them and why?..............................................

.

AC15: Did you enjoy any input subsidies from government in the 2018 farming season?

AC16: If yes to AC15, on which inputs, ....................................................................................................................

AC17: If yes to AC15, how much per input?...............................................................................................................

AC18: If yes to AC15, what was the repayment plan?.......................................................................................

## Section Q: Access to Insurance

AI1. Are you aware of agricultural insurance? 1=Yes 0= No

AI2. If yes, have you ever purchased insurance products to cover your maize farm? 1=Yes 0= No

AI3. If No to **AI2**, why have you not purchased insurance product? 1=Lack of funds, 2=No idea of how it works, 3=It doesn't pay, 4.Other, specify_____________

**If you have ever purchased an insurance product please answer the following questions**

AI4. What type of product did you purchase?......... 1=Area yield; 2=Multi-Peril; 3=Drought Index

AI5. Give reasons why you purchased this product(s)?..............................................................

AI6. Which year did you purchased your first product? _______

AI7. If you purchased insurance product(s) in the previous farming season, did you repeat the purchase in the subsequent seasons? 1=Yes 0=No

AI8. If Yes to **AI7**, why did you repeat the purchase? 1=Had pay-out when I bought the insurance previously; 2=Had a good harvest; 3=Other_________

AI9. If No to A**I7**, why did you not repeat the purchase in the subsequent seasons? 1. Did not get a pay-out when I suffered a loss; 2.High premium rate; 3. Lack of money; 4. I didn't see the benefits of having insurance 5. Other_____________

AI20. Which other crop did you purchase insurance to cover?......1=Rice; 2=Sorghum; 3=Millet; 4=Others, specify………………

**Section R: Social Networks**

**R1: Relationship and characteristics**

Please I want to understand your relationship and interactions with farmers in this community/village.

| Farmers | HH ID | Do you know (X)? | Since when (year) have you known (X)? | What is the age of (X)? (in years) | What is the educational level of (X)? (in years) | In what way is (X) related to you? *(Relationship code)* | Are you in the same religious congregation with X? | Are you in the same association with (X)? *See code below* | Have you ever spoken to (X)? 1=Yes 2=No | If SN9 is yes, how often within a month on average do you speak to (X)? | Have you ever been to the house of (X)? | If SN9 is yes, how many times within a month do you visit the house of (X)? | If SN9 is yes, what is the distance between the HH | Is (X)'s farm adjacent to yours? | Have you ever passed by the farm of (X)? | If yes to SN12, how often within a month do you pass by (X)'s farm | Does X interact with village officials? |
|---|---|---|---|---|---|---|---|---|---|---|---|---|---|---|---|---|---|
| | **SN1** | **SN2** | **SN3** | **SN4** | **SN5** | **SN6** | **SN7** | **SN8** | **SN9** | **SN10** | **SN11** | **SN12** | **SN13** | **SN14** | **SN15** | **SN16** | **SN17** |
| | | **Farmers from same /community/village** | | | | | | | | | | | | | | | |
| 1 | | | | | | | | | | | | | | | | | |
| 2 | | | | | | | | | | | | | | | | | |
| 3 | | | | | | | | | | | | | | | | | |
| 4 | | | | | | | | | | | | | | | | | |

**Relationship Codes** 1=Parent; 2=Child; 3=Brother/sister; 4=Grandparent; 5=Grandchild; 6=Nephew/Niece; 7=Uncle/aunt; 8=Cousin; 9=Same family lineage; 10=Mother/father in-law; 11=Brother/sister in-law;12=Other relative; 13=Fellow villager/Neighbour; 14=Attend same church/ mosque; 15=Professional/business colleague; 16=Other, specify…….
**Codes SN8**: 0=No; 1=FBO; 2=Self-help group; 3=Merry go round; 4=Savings and Credit; 5=Other (Specify)………… **Codes SN17** 1= No, 1=chief, 2=assemblymen/women, 3=village/committee chairman, 4=others specify…………

**R2: Information sharing and adoption process**

| Farmers | HH ID Do you discuss/ Share SASs issues with X? | Does (X) inform you of upcoming extension meetings | Who first told you about sustainable agricultural strategies? (refer to section D for examples of SASs)? | Who convinced you to adopt any of the sustainable agricultural strategies? | When did X adopt the SASs? (years) | Do you exchange more than farming information with X? 1=Yes, 2=No | If yes to SN6, which other information do you share? | How many blood relatives do you have in the community | Do you belong to a royal family | Are/were your parents leaders? |
|---|---|---|---|---|---|---|---|---|---|---|
| | **SN1** | **SN2** | **SN3** | **SN4** | **SN5** | **SN6** | **SN7** | **SN8** | **SN9** | **SN10** |
| **Farmers from same village** | | | | | | | | | | |
| 1 | | | | | | | | | | |
| 2 | | | | | | | | | | |
| 3 | | | | | | | | | | |
| 4 | | | | | | | | | | |

**Code SN5**: 1=Not yet, 2=Before me, 3=At the same time as me, 4=After me **Code SN7**: 1=credit, 2=labour, 3=marketing, 4=access to inputs, 5=insurance, 6=others specify…………

## Section S. Shocks and coping mechanisms

Please list all shocks that hit the household during the past 2 years (2017 and 2018).

| | S.0 Shock | S.1. Did the HH experience any of the following shocks in the last 2 years? 1=yes; 2=no | S.2. If S1 is yes, How many times did it occur in the last 2 years? | S.3 If S1 is yes, which year did it occur? (month/ year) | S.4 How much time did it take to economically recover from the shock? 1. < 1 month 2. 1-3 months 3. 4-6 months 4. 7 months-1 year 5. > 1 year 6. Not yet recovered | S.5 What is the likelihood that these shocks will happen again (1-2 years)? 1. Very likely 2. A bit likely 3. Fifty/fifty 4. Not so likely 5 Completely unlikely 6. I don't know | |
|---|---|---|---|---|---|---|---|
| **1** | Flooding | | | | | | |
| **2** | Long dry spells | | | | | | |
| **3** | Erratic rainfall pattern | | | | | | |
| **4** | Windstorm | | | | | | |
| **5** | Pest and disease | | | | | | |
| **6** | Livestock disease | | | | | | |
| **7** | Bushfire | | | | | | |
| **8** | Increase in prices of farm inputs | | | | | | |
| **9** | Very low market/farm gate price for produce | | | | | | |
| **10** | High unemployment | | | | | | |
| **11** | Sickness of household member | | | | | | |
| **12** | Death of household member | | | | | | |
| **13** | Very low yields | | | | | | |
| **14** | Crop failure | | | | | | |
| **15** | Others, specify | | | | | | |

**S.6 How did your HH deal with the shocks?**
Please list (4) in order of importance

1. Prayed to God
2. Hardworking
3. Diversification
4. crop diversification
5. Inputs substitution
6. Reduction in HH expenditure
7. Use of weather information

7. Took children out of school
8. Sent children to relatives/friends
9. Adult household member migrated
10. Sold assets
11. Used savings

12. Purchased insurance product
13. Borrowed money from family/ friends/ neighbors
14. Borrowed money from informal financial institution (pawnshop, moneylender, susu collectors, traders)
15. Borrowed from formal financial institution (bank, microfinance)
16. Government/NGO support
17. Support from social networks
18. Other, specify

| S6a | S6b | S6c | S6d |
|---|---|---|---|
| | | | |

| | | | |
|---|---|---|---|
| | | | |

**S.7. What measures have you (HH) put in place to reduce or prevent these shocks if they reoccur?**
Please list 4 in order of importance

1. Prayed to God
2. Hardworking
3. Diversification
4. crop diversification
5. Inputs substitution
6. Reduction in HH expenditure
9. Hold savings at formal institution (bank, microfinance)
10. Hold savings at informal institution (Susu, etc.)
11. Participate in network activities
12. Purchase insurance
13. Other, specify

| **S.7.a** | **S.7.b** | **S.7.c** | **S.7.d** |
|---|---|---|---|
| | | | |

## Section T: Perception of Rainfall Variability

T1. What was the **rainfall variability** over the last 3 farming seasons **(2017, 2016, and 2015)**? 1=highly favourable 2=Favourable, 3= Unfavourable, 4=Highly unfavourable

T2. What was the **amount of rainfall (intensity)** over the last 3 farming seasons (2017, 2016, and 2015)? 1=Low, 2=Moderate, 3=High

T3. What was the **rainfall variability** during the 2018 farming season? 1=highly favourable 2=Favourable, 3= Unfavourable, 4=Highly unfavourable

T4. What was the **amount of rainfall (intensity)** during the 2018 farming season? 1=Low, 2=Moderate, 3=High

T5. Is the **annual distribution** of the rainfall on your farm favourable? 1=yes, 2=No
