# Supplementary material for: Minimum tillage as climate-smart agriculture practice and its impact on food and nutrition security
Source: PLoS One. 2023 Dec 22;18(12):e0287441. doi: 10.1371/journal.pone.0287441 (PMC10745223; doi:10.1371/journal.pone.0287441)
Supplement: S1 Appendix — (DOCX) [file pone.0287441.s003.docx]

| **Table A1: Test of validity of selection instruments** | | | | |
| --- | --- | --- | --- | --- |
| **Variable** | **Maize**  **yields** | **HFIAS** | **Dietary diversity** | **Labor demand** |
| Climate information | 0.07 | 0.16 | -0.11 | 0.03 |
|  | (0.06) | (0.37) | (0.28) | (0.08) |
| CA Training | 0.03 | 0.20 | -0.13 | -0.004 |
|  | (0.02) | (0.14) | (0.16) | (0.04) |
| F-test on instruments | 1.79 | 1.06 | 0.56 | 0.05 |
| *p*-value | 0.17 | 0.57 | 0.54 | 0.95 |

| **Table A2. Disaggregation of labor demand** | | | |
| --- | --- | --- | --- |
| **Variable** | **Description** | **Mean** | **Std. Dev** |
| Labor demand | Total person-days per acre | 33.77 | 27.44 |
| Hired labor | Hired labor (person-days) used per acre | 16.60 | 19.27 |
| Family labor | Family labor (person-days) used per acre | 17.17 | 12.28 |
| Land preparation | Labor (person-days/acre) used for land preparation | 5.08 | 3.80 |
| Planting | Labor (person-days/acre) used for planting | 5.49 | 5.53 |
| Weeding | Labor (person-days/acre) used for weeding | 8.02 | 8.25 |
| Harvesting | Labor (person-days/acre) used for harvesting | 5.80 | 4.70 |
| Threshing | Labor (person-days/acre) used for threshing | 3.87 | 2.54 |
| Herbicide application | Labor (person-days/acre) used for herbicide application | 2.70 | 2.76 |
| Fertilizer application | Labor (person-days/acre) used for pesticide application | 2.81 | 2.21 |
|  | | | |

**Table A3: APPENDIX B: correlation between instrumental variable outcomes variables**

| **Instrument** | **Climate information** | | **Training** | |
| --- | --- | --- | --- | --- |
| **Dependent variable** | Correlation | *p*-value | Correlation | *p*-value |
| Maize yield | 0.1248 | 0.0955 | 0.0553 | 0.2751 |
| HFIAS | -0.0038 | 0.9397 | -0.0746 | 0.1407 |
| Dietary diversity | 0.0589 | 0.2455 | 0.0112 | 0.8256 |
| Labor demand | 0.0038 | 0.9405 | 0.0324 | 0.5234 |

**Table A4. First-stage regressions of the IV-GMM**

| **Variables** | **First-stage IV-GMM** | |
| --- | --- | --- |
|  | Coefficient | S.E. |
| Age | 0.002 | 0.002 |
| Male headed | 0.161*** | 0.044 |
| HH size | -0.003* | 0.007 |
| Education | 0.008 | 0.005 |
| FBO | 0.117*** | 0.044 |
| Farm size | 0.019** | 0.009 |
| Extension | 0.014*** | 0.004 |
| Credit access | -0.07 | 0.043 |
| Livestock | 0.001 | 0.006 |
| Mean_slope | -0.170*** | 0.043 |
| Mean_fertile | 0.048 | 0.058 |
| Mean_Mod. fertile | 0.122** | 0.055 |
| Asset (log) | 0.163*** | 0.018 |
| Negative rainfall shock | 0.117 | 0.073 |
| Mean temperature | -0.255*** | 0.053 |
| Fertilizer (log) | -0.066*** | 0.014 |
| Climate information | 0.244*** | 0.048 |
| CA Training | 0.099*** | .012 |
| Northern | -0.371*** | 0.070 |
| Upper West | -0.199*** | 0.089 |
| Constant | 7.329 | 1.459 |
|  |  |  |
| R^2^ | 0.921 |  |
| Weak identification tests: |  |  |
| Cragg-Donald F-statistic | 257.63*** |  |
| Kleibergen-Paap rk Wald F statistic | 77.75 |  |
| P-value of Angrist-Pischke F-test | 0.0000 |  |
| Over identification test: |  |  |
| Hansen J | 1.204 |  |
| *p*-value | 0.2726 |  |

*NB:* the table presents firsts-stage estimations of the IV-GMM regression of length of adoption on the main set of controls and the instruments as in our main model reported in Table 2.
